# Hybrid Metal Catalysts as Valuable Tools in Organic Synthesis: An Overview of the Recent Advances in Asymmetric C─C Bond Formation Reactions

**DOI:** 10.3390/molecules29215090

**Published:** 2024-10-28

**Authors:** Isabella Rimoldi, Giulia Coffetti, Raffaella Gandolfi, Giorgio Facchetti

**Affiliations:** Department of Pharmaceutical Sciences, University of Milan, Via Venezian 21, 20133 Milano, Italy; isabella.rimoldi@unimi.it (I.R.); giulia.coffetti@unimi.it (G.C.); raffaella.gandolfi@unimi.it (R.G.)

**Keywords:** artificial metalloenzymes, metallo peptides, homogeneous catalysis, organometallic complexes, protein engineering, enantioselective C─C bond formation

## Abstract

Carbon–carbon bond formation represents a key reaction in organic synthesis, resulting in paramount importance for constructing the carbon backbone of organic molecules. However, traditional metal-based catalysis, despite its advantages, often struggles with issues related to efficiency, selectivity, and sustainability. On the other hand, while biocatalysis offers superior selectivity due to an extraordinary recognition process of the substrate, the scope of its applicable reactions remains somewhat limited. In this context, Artificial Metalloenzymes (ArMs) and Metallo Peptides (MPs) offer a promising and not fully explored solution, merging the two fields of transition metal catalysis and biotransformations, by inserting a catalytically active metal cofactor into a customizable protein scaffold or coordinating the metal ion directly to a short and tunable amino acid (Aa) sequence, respectively. As a result, these hybrid catalysts have gained attention as valuable tools for challenging catalytic transformations, providing systems with new-to-nature properties in organic synthesis. This review offers an overview of recent advances in the development of ArMs and MPs, focusing on their application in the asymmetric carbon–carbon bond-forming reactions, such as carbene insertion, Michael additions, Friedel–Crafts and cross-coupling reactions, and cyclopropanation, underscoring the versatility of these systems in synthesizing biologically relevant compounds.

## 1. Introduction

A chemical reaction cannot occur unless its activation energy is overcome, regardless of its energetically favorable profile. While some spontaneous reactions can easily overcome this energetic barrier, in most situations, the transformation is unfavorable and relies on the use of a catalyst, i.e., a substance able to reduce the activation energy of a reaction by modifying the transition states of the reactants, without itself being consumed or irreversibly altered but, conversely, quickly being regenerated during the process [1,2,3]. Given the ever-increasing demand for non-racemic chiral compounds, the development of efficient methodologies for the production of enantiomerically pure compounds is of paramount importance across both the industrial field and academia [4,5]. Transition-metal-based catalysts play a pivotal role in this context, offering unique potentiality in the isolation of pure enantiomers through asymmetric catalysis [6,7]. Over recent decades, catalysis has become a central focus among Green Chemistry principles, leading to a shift from inefficient stoichiometric methods to more sustainable, catalytic alternatives. Among the various strategies available, asymmetric catalysis, stands out as one of the most versatile approaches, ensuring both high stereoselectivity and reduced environmental impact.

There are three main types of catalysts, namely, homogeneous catalysts, heterogeneous catalysts, and biocatalysts (enzymes). Although homogeneous systems are typically non-reusable, they are characterized by well-defined active sites, allowing for a wide range of structural optimization leading to outstanding performances both in terms of conversion and selectivity [8]. In contrast, heterogeneous systems tend to be less structurally uniform, and sometimes they could require more complex synthesis processes to be achieved. However, heterogeneous catalysts are reusable, easier to separate from the desired products, and, most importantly, they could offer the possibility of developing continuous processing [9,10]. Both homogeneous and heterogeneous reactions still have to face practical limitations, including substrate scope limitations, stability, and solvent compatibility. On the other side, most organic chemists have been reluctant to include enzymes in their toolbox due to traditional limitations such as the narrow substrate scope and limited repertoire of reactions [11]. Conversely, transition metal catalysts demonstrated their versatility in a broad range of challenging transformations including C-H activation [3,12,13], metathesis reactions [14,15,16], and asymmetric transfer hydrogenation in a more sustainable way [17,18,19]. In such catalysts, the presence of easy-to-modulate ligands around the metal center provides the possibility to properly tune the reactivity of the metal ion, thus generating chemoselective catalysts able to interact with specific substrates. The rational design of the ligand structure, by introducing selected substituents or their production in an enantiopure form, could be in principle a valid approach to impart stereo- and site selectivity to the catalyzed reaction [20,21]. Similarly to natural enzymes, the introduction of hydrophilic substituents makes them compatible with the aqueous reaction environment [22,23], a feature particularly attractive for the development of greener chemical reactions [24,25].

**Figure 1:**
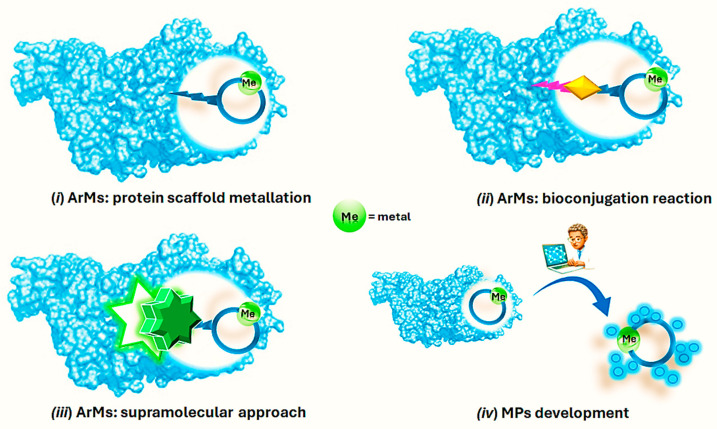
Schematic representation of the different approaches for the development of ArMs and MPs.

Within the context of a more sustainable catalysis, Artificial Metalloenzymes (ArMs) have stemmed from a fascinating idea to merge the exquisite substrate selectivity exhibited by natural enzymes with the wide repertoire of transformations relying on transition metal catalysis [26,27,28,29]. The so-formed hybrid catalysts benefit from both the reactivity of the metal-based catalyst, i.e., the first coordination sphere, and the well-defined chiral environment furnished by the protein secondary coordination sphere that is able to induce selectivity to the metal ion, furnishing a system with a new-to-nature ability of carrying out organic transformations [27,30]. At least three different anchoring strategies enable the formation of such hybrid catalysts and ensure a robust insertion of the metal cofactor within the protein scaffold. These can be broadly summarized as *(i)* ArMs based on protein scaffold metalation involving specific natural amino acids (Aas); *(ii)* ArMs originating from the covalent attachment of a synthetic and catalytically active transition metal complex to a suitable protein through a bioconjugation reaction; *(iii)* ArMs relying on an extensive array of supramolecular interactions occurring between the metal cofactor and the protein directly or with an anchor moiety for which the protein shows high affinity [27,29,31,32,33,34] (Figure 1).

Along with the use of protein scaffold as a secondary coordination sphere, the use of small peptides up to 50 Aas that are able to bind transition metals in the corresponding Metallo Peptide catalysts (MPs, Figure 1) *(iv)* has recently emerged as a valid alternative in the preparation of hybrid catalysts because of the easiness of small peptides’ synthesis if compared to protein expression [35,36,37,38]. By rationally modulating the Aa sequence, the peptide complexation ability as well as the catalyst performance could be modulated, thus gaining access to different and otherwise difficult achievable chemical reactions [39]. Finally, considering the molecular recognition properties offered by peptides, it is possible to tune the second coordination sphere of the metal by introducing supramolecular interactions such as β-turns. 

This review would offer an overview about hybrid catalysts’ development focused on the most original and recent advances (from 2018 up to now) in both ArM and MP systems applied to the asymmetric C─C bond formation reactions. 

## 2. Different Hybrid Catalysts in C─C Bond Formation Reactions

### 2.1. Hybrid Catalysis Applied to Carbene Insertion Reactions

Carbene insertion into C─H bonds provides an attractive and versatile synthetic approach for the formation of new C─C bonds, an essential reaction for increasing the complexity of organic molecules [40,41,42,43]. 

**Scheme 1:**
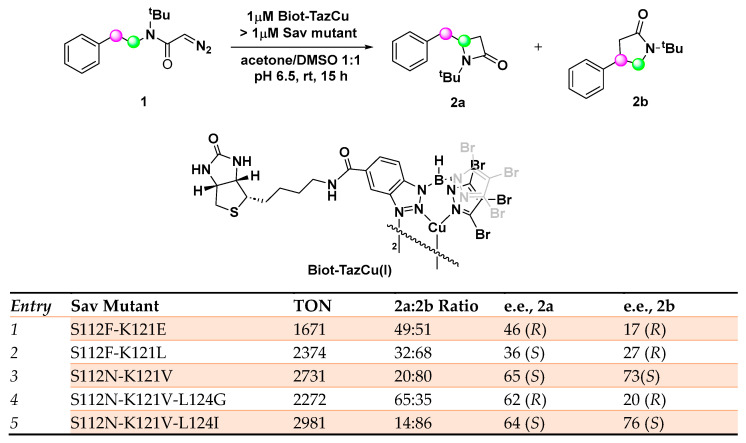
**Reaction conditions:** reactions were carried out using 5 mM of **1**, 0.02 mol% of Biot-TazCu, >0.02 mol% of Sav mutant, acetone 5%, DMSO 5%, pH 6.5, rt, 15 h [44].

An efficient artificial carbene insertase has been recently developed by incorporating a biotinylated copper(I) heteroscorpionate complex (Biot-TazCu) within streptavidin (Sav) endowed with catalytic activity in the regio- and enantioselective intramolecular insertion of diazocetamide into sp^3^ C─H bonds with the formation of important building blocks such as those of β- and γ-lactams [44,45]. Tris(pyrazolyl)borate (Tp) as coordinative ligand was rationally selected for the synthesis of the metal cofactor in virtue of the possibility to finely tune the steric and the electronic properties, thus achieving the desired reactivity [46]. The biotin–streptavidin technology is a versatile tool for the preparation of robust ArMs relying on the remarkable affinity of biotin for streptavidin (K_d_ < 10^−13^ M) [47,48,49]. Computation studies highlighted S112, K121, and L124 as the residues of the host protein crucial for the outcome of the reaction. Through a double saturation mutagenesis optimization, a library of 400 Sav isoforms Sav S112X-K121X among which 220 afforded detectable levels of activity. This strategy revealed the necessity of an apolar side chain at position K121 in order to obtain an efficient carbene insertase, whereas a strong difference could be detected between aspartate and its homologous glutamate with the second one proving beneficial for the ArM activity when occupying both positions. If positively charged residues such as His, Lys, and Arg at position K121 resulted in being detrimental for the activity, an asparagine residue at position S112 appeared to be crucial for the enantioselective synthesis of both β- and γ-lactams. As a result of this double saturation mutagenesis study, the S112N-K121V variant was identified as the most promising, both in terms of activity and selectivity, leading to 20:80 e.r. in favor of **2b** with 73% e.e. and 2731 TON. Starting from these premises, a second round of directed evolution was then realized focusing on residue Sav L124 whose steric hindrance strongly impacted the **2a**/**2b** ratio and e.e. The mutation of L124 with an isoleucine slightly increased the selectivity of the ArM for the γ-lactam **2b** (76% e.e. (*S*)), while a glycine residue favors the β-lactam **2a** (62% e.e. (─., identified as the (*R*) enantiomer by VCD experiments) (Scheme 1). 

**Scheme 2:**
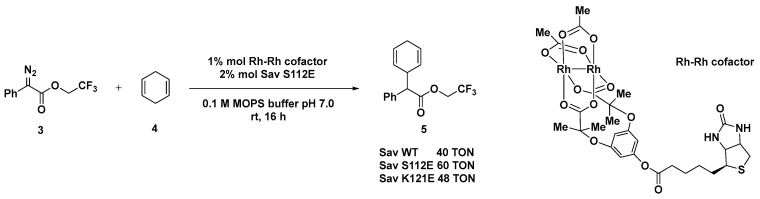
**Reaction conditions:** reactions were carried out using 10 mM of **3**, 50 mM of **4**, 1 mol% Rh complex, 2 mol% Sav mutant, 0.1 M MOPS buffer pH 7.0, rt, 16 h [51].

The same protein scaffold was exploited by the same group for the preparation of ArMs based on dirhodium complexes [50,51], known for being efficient catalysts in carbene insertion reactions [51]. This class of artificial carbene transferase was developed by applying both a chemical optimization of the metal cofactor structure and a genetic engineering of the Sav mutants. Chiral isoindolones were synthesized starting from N-(pivaloyloxy)benzamides and aromatic diazoesters bearing different functionalities such as biphenyl, thioether, selenoether, amine, olefin, and alkyne, with up to 90% e.e. Crystal structure studies of the biotinylated Rh(III) cofactor ⊂ Sav indeed highlighted position N49 as crucial in determining the enantiodivergence of the hybrid system. Computational studies [52], however, showed that the dirhodium cofactor protrudes out of the biotin-binding pocket, thus accounting for the modest effect provided by the mutagenesis experiments if compared to the chemical modification upon the rhodium tetracarboxylate catalyst. Sav S112E and K121E demonstrated the highest efficiency in the C─H insertion of trifluroethyl(phenyl) diazoacetate **3** with 1,4-cyclohexadiene **4** with TONs up to 60, although any enantioselectivity could not be detected (Scheme 2). The same hybrid system was also successfully applied to the cyclopropanation reaction of styrene with ethyl diazoacetate or the donor–acceptor ethyl(phenyl)acetate. The authors reported a mutagenesis study of the Sav in order to increase the TON and the selectivity, but, unfortunately, also in this case the incorporation of the biotinylated dirhodium cofactor within Sav did not show any effect on either the diastereoselectivity (*trans*/*cis* ratio up to 1.5/1 with Sav WT) or the enantioselectivity (racemic product). The introduction of basic Aas in proximity of dirhodium cofactor, such as S112C, S112H, or K121D, led to comparable TONs to Sav-WT (up to 60). When the reaction was conducted in the presence of the bulkier donor–acceptor diazo(phenyl)-acetate, considerably lower TONs were achieved with the exception of some Sav mutants bearing Lewis-basic side chains (S112D, S112K, and S1112H). 

**Scheme 3:**
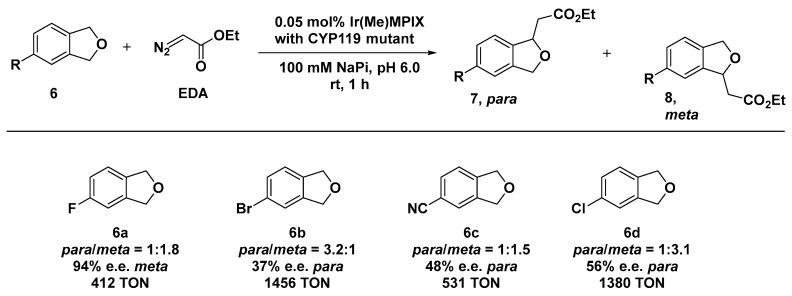
**Reaction conditions:** reactions were carried out using 5mM of **6**, 0.05 mol% of Ir(Me)MPIX⊂CYP119 MG_1_ (**6a**) or Ir(Me)MPIX⊂CYP119 PG_2_ (**6b**, **6c,** and **6d**), 100 mM NaPi (sodium phosphate buffer) pH 6.0, rt, 1 h [58].

Within the same field, Hartwig and co-workers explored the use of Cytochrome P450 variant CYP119 as an effective scaffold for the preparation of artificial carbene insertases by incorporating an Ir(Me) cofactor (here, Me stands for methyl) [53,54]. Heme proteins have been extensively investigated as biocatalysts for carbene insertion reactions upon the substitution of the iron center with non-native metal ions [55,56,57]. The ArM insertases were obtained by formally replacing Fe with an Ir(Me)-mesoporphyrin IX (Ir(Me)-MPIX) cofactor along with four C317G, T213G, L69V, and V254L mutations in CYP119 by directed evolution method. The obtained hybrid systems displayed remarkable versatility and a wide substrate scope, comprising insertion into activated and inactivated C─H primary bonds and sterically hindered ones. The CYP119 library was constructed from the double-mutant enzyme containing the T213G and C317G mutations to which additional mutations were introduced in the residues closely lying by the catalytic site. The generated systems catalyzed the insertion of carbenes into the C─H bonds of a range of phthalan derivatives **6** containing substituents that render the two methylene positions in each phthalan inequivalent [58]. The system resulted in being effective, even when applied to the intermolecular insertion of ethyldiazoacetate (**EDA**) into either a *para*- or *meta*-benzylic C─H bond of phtalan, which afforded the corresponding products in appreciable diastereomeric excess (52% d.e. for the *para-* and 55% d.e. for the *meta*-substituted product). Beyond site selectivity, some of the reactions did occur with a striking enantioselectivity. For example, when the variant MG1 + P252S (*meta* generation 1: C317G, T213G, V254A, A152L + single mutation P252S) was applied to 4-fluorophtalan (**6a**, Scheme 3), the insertion reaction afforded the major formed isomer in 94% e.e. and the minor isomer in 84% e.e. with a 1:1.8 ratio of constitutional isomers in favor of the *meta* one. 

By combining directed evolution experiments and computational studies, it has been demonstrated that mutations L69V and V254L selectively introduced at the distal positions, with respect to the catalytic site, could significantly increase the achievable TONs thanks to the precise orientation of the substrate cofactor within the host protein and by the flip of the cofactor from an inactive to an active conformation [59]. This activation process resulted in a net stabilization of the transition state and in lowering the activation energy barrier for the rate determining step of iridium–porphyrin–carbene intermediate (Ir-PC), accounting for the enhanced catalytic activity of the system. Upon the N_2_ group leaving, it can be revealed that this unusual stabilization in the transition state of the vacant *p* orbitals at the carbene center was due to the backdonation of π electrons from the aryl ring [59,60]. This effect could be particularly appreciated with more electron-rich substrates, postulated to be able to surpass even the natural enzymes in terms of catalytic rate enhancement [60]. When applied to the synthesis of methyl 2,3-dihydrobenzofuran-3-carboxylate, the reaction catalyzed by the artificial Ir(Me)-CYP119 showed astonishing TON exceeding 3200 and 93% e.e. even for Gram-scale production. 

### 2.2. Michael Addition Reactions Catalyzed by Hybrid Catalysts

**Figure 2:**
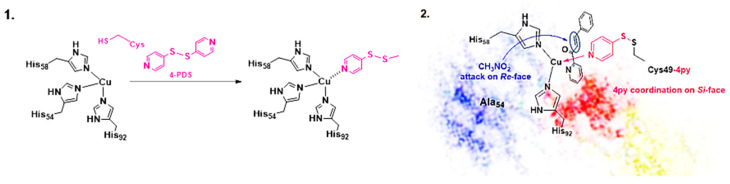
(**1**) Cysteine modification with 4-PDS to form Cys-4py as ligand for Cu(I) coordination; (**2**) Substrate binding to the copper centers in I49C-4py/H52A/H54A/C106D to reverse the enantioselectivity of the artificial system affording (*R*)-form. Reprinted from [64] Copyright (2024), with permission from Elsevier.

**Scheme 4:**
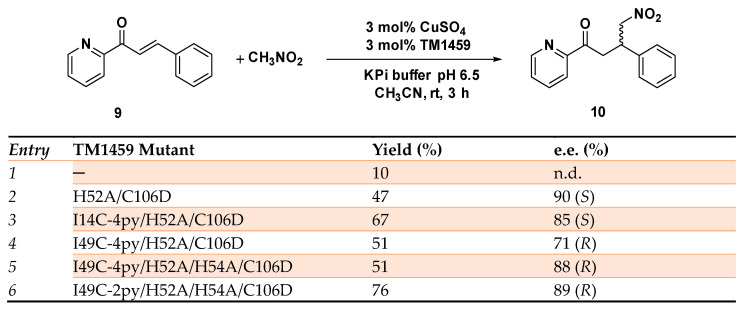
**Reaction conditions:** reactions were carried out using TM1459 0.3 mM, CuSO_4_ 0.3 mM, substrate **9** 10 mM, CH_3_NO_2_ 100 equiv., KPi (potassium phosphate buffer) pH 6.5/CH_3_CN 9:1, rt, 3 h [64].

The Michael addition reaction provides one of the most versatile C─C bond formation synthetic approaches, both in the laboratory and at industrial level [61,62]. The reaction involves a nucleophile as a Michael donor and an activated olefin playing as a Michael acceptor giving rise to the formation of a new covalent bond. The use of heterocycles as donors has paved the way for the synthesis of valuable compounds in medicinal as well as in the industrial field [63]. Recently [64,65], the Fujieda group reported the synthesis of a non-heme metalloenzymes using a cupin superfamily protein (TM1459) able to enantioselectively catalyze the Michael addition of nitromethane to α,β-unsaturated ketone, 2-aza-chalcone 9 (Scheme 4). The protein, known for possessing a metal binding site comprising four histidine residues, is a naturally Mn-binding protein, but its osmium-based Artificial Metalloenzyme has already been reported by the same group in the regioselective *cis*-1,2-dihydroxylation reaction [66]. Both H52A and H52A/C106D copper mutants showed excellent (*S*)-enantioselectivity in the Michael addition of nitromethane to azachalcone with the H52A/C106D affording the highest yield and (*S*)-enantioselectivity up to 90% e.e. The solved crystal structure of the Cu-bound H52A mutant provided important insights relative to the metal binding site, showing a His triad (His54, His58, and His92, Figure 2) around the coordinated copper ion and the hydrophobic pocket for substrate allocation *trans* to His54. The nitromethane attack to the *Si*-face of the azachalcone was found to be responsible for the preferential (*S*)-enantiomer formation (Figure 2). By properly modifying the metal binding site in order to obtain a mirror-like image of the coordination structure of H52A, a reversal of the enantiopreference was achieved in favor of the (*R*)-enantiomer. Due to their proximity to the copper center, Ile14, Lys18, Lys24, and Ile49 were identified for selectively introducing a Cys residue to be successively reacted with 4-PDS (4,4′-dithiopyridine). ESI-MS was recorded to prove that nearly all site-directedly introduced Cys residues were effectively modified by 4-PDS to produce the corresponding Cys-4py moiety in the mutants. The substitution of isoleucine 49, indeed, showed the most significant impact on the enantioselectivity of the reaction that proved extremely sensitive to the steric hindrance of the Aa residue introduced. The I49C4py/H52A/C106D mutant afforded the product in 71% e.e. of the (*R*) product, experimentally confirming the reversal of enantiopreference. To improve the (*R*)-enantioselectivity, a second histidine mutation was introduced into I49C-4py/H52A/C106D to construct a coordination structure with mirror symmetry to the active site of H52A. Accordingly, the I49C-4py/H52A/H54A/C106D mutant was prepared, displaying an increase in the enantioselectivity up to 88% e.e. (*R*) but in 51% yield. 

The subsequent modification with 2,2′-dithiopyridine (2-PDS) instead of 4-PDS allowed us to isolate the Michael addition reaction product with comparable enantioselectivity but with higher yield (76% yield, 89% e.e. *R*), as a result of the beneficial 2-PDS modification on the coordination site around the copper center (Scheme 4). This work reported for the first time an ArM with reversed enantioselectivity in the Michael addition reaction due to the rational design of the first coordination sphere. [64]

Advances gained in computational design and the understanding of peptide stability and folding behavior have greatly expanded the range of customizable peptide sequences [36]. The possibility to introduce specific natural and non-natural Aas or/and to synthetically modify both the backbone and side chains [67,68] showed to dramatically impact the conformational primary structure with evident consequences on the peptide folding and functionality. Many essential biochemical processes rely on proteins characterized by the presence of a metal cofactor to display their functions. Thus, taking inspiration from nature, peptides can be used to mimic enzyme active sites by re-designing their former structure with coordinative features able to host non-native metal ions, making the resulting hybrid systems (MPs) original duplicates endowed with new-to-nature activities [69]. 

**Figure 3:**
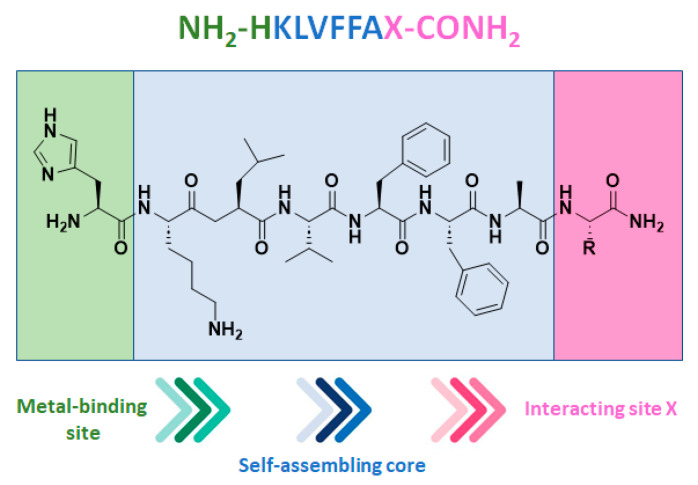
Structure of the peptide sequence comprising the self-assembling Aβ amyloid core developed as new coordination spheres for Cu(II) hybrid catalysts. Reproduced from Ref. [72] with permission from the Royal Society of Chemistry.

Starting from the core sequence of Aβ amyloid peptide (KLVFFA), known for being responsible of neurodegenerative diseases [70,71], and altering the N- and C-terminal residues, new coordination spheres for metal ions have been developed and evaluated as copper(II) hybrid catalysts in terms of the asymmetric Michael additions between 2-azachalcone and dimethyl malonate (**DMM**) [72]. At the N-terminus, a histidine residue was introduced as a metal-binding residue, whose primary amino group and imidazole side chain could be exploited for metal coordination (Figure 3). 

**Scheme 5:**
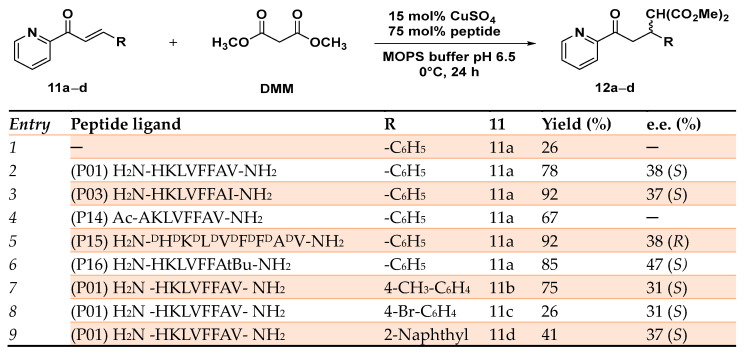
**Reaction conditions:** reactions were carried out using 475 µM of peptide, CuSO_4_ 95 µM in 20 mM MOPS buffer pH 6.5, 0 °C, 24 h [72].

This pattern is extremely diffuse in natural enzymes such as polysaccharide monooxygenases [73] and in the bacterial membrane protein methane monooxygenase [74]. By selectively modifying the C-terminal residue with different Aas able to differently interact with the substrate, 12 self-assembling peptides (P01–P12) were synthesized. The corresponding copper(II)-based MPs showed moderate to appreciable enantioselectivity (up to 47% e.e. (*S*) for the P16 H_2_N-HKLVFFAtBu-NH_2_) and good to excellent yields (up to 92% for the P03 H_2_N-HKLVFFAI-NH_2_). The obtained results demonstrated that when an aliphatic residue was introduced at the C-terminal (Val, Leu, Ile, and Met), the resulting MPs catalyzed the C─C bond formation with better enantioselectivity and yield. Conversely, the bulky aromatic Aas Tyr and Trp were responsible for a significant erosion of the catalyst activity. Similar results were obtained even when the artificial MP was applied to the Michael addition reaction of differently substituted azachalcones **11a**–**d**, showing the robustness and versatility of the system (Scheme 5). 

When the N-terminal histidine was acetylated (P14 Ac-AKLVFFAV-NH_2_) or replaced by a different residue lacking a suitable side chain able to coordinate the metal center, the reaction proceeded with a drastic erosion of enantioselectivity, thus demonstrating the stabilization effect on metal coordination favored by the N-terminal histidine. Moreover, the enantioselectivity of the reaction could be reversed from the preferred (*S*) product affording the opposite (*R*) enantiomer by simply employing D-amino acids for the synthesis of the peptide ligand (P15 H_2_N-^D^H^D^K^D^L^D^V^D^F^D^F^D^A^D^V-NH_2_) in place of the natural ones (Scheme 5). The authors demonstrated by FT-IR and CD analyses that the enantioselectivity and the reactivity of such MPs were due to the self-assembly behavior of the synthesized peptides upon coordination to the copper center and their folding in antiparallel β-sheet structures. 

**Scheme 6:**
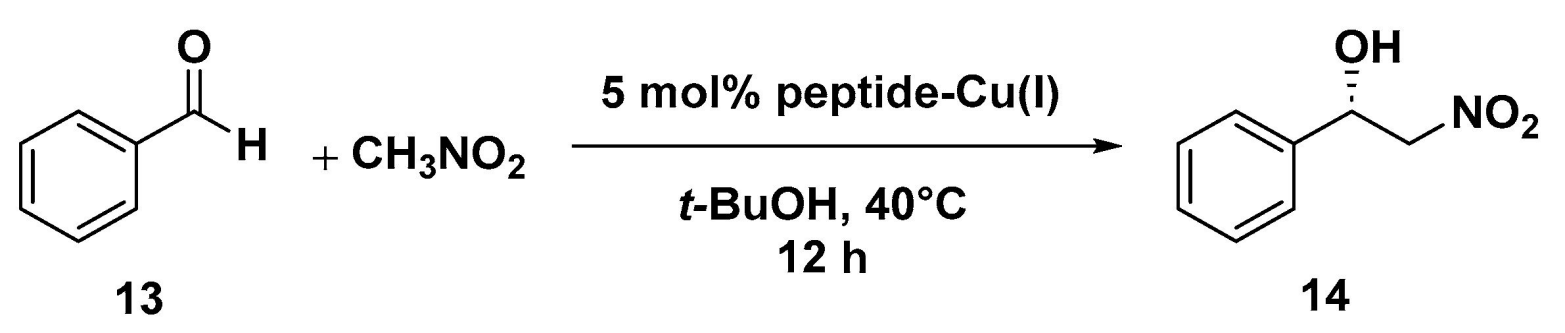

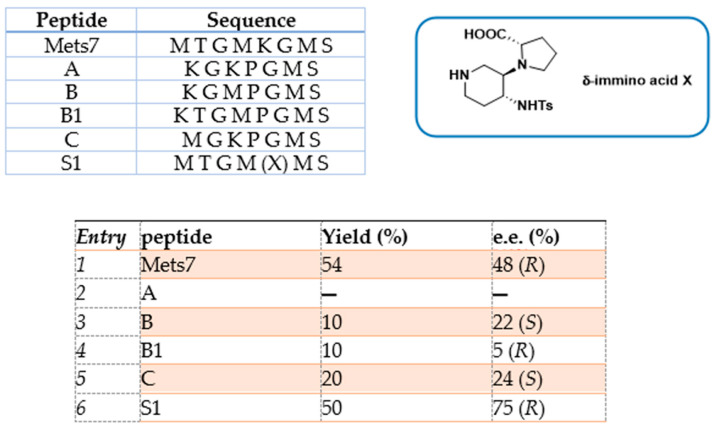
**Reaction conditions:** reactions were carried out using 5 mol% (0.05 equiv.) of peptide-Cu(I) complex in *t*-BuOH:CH_3_NO_2_ = 50:50 (2 mL), substrate **13** 1.0 equiv., at 40 °C, 12 h [76].

The asymmetric Michael addition of nucleophiles to chalcone substrates represents a valuable synthetic strategy for the preparation of useful intermediates of pharmaceutical interest, such as GABA receptor agonists and MAOB (monoamine oxidase type B) inhibitors for the treatment of neurodegenerative disorders [75]. In a previous work by our group, we reported the use of octapeptide Mets7 and its derivatives as ligands for the preparation of a small library of copper(I)-based catalysts for the enantioselective Henry condensation reaction [76]. Mets7 is a methionine-rich motif naturally expressed at the N-terminal of the human Copper transporter 1 (hCtr1) involved in the physiological trafficking of copper across the membrane but also established as being involved in platinum chemotherapeutics resistance phenomena [77,78]. When the Mets7 sequence was rationally modified by introducing the unnatural scaffold X, i.e., a δ-amino acid able to stabilize β-structures when inserted in model peptides [79], the corresponding Cu(I) complex afforded the nitromethane condensation reaction product **14** with benzaldehyde in 75% e.e. (*R*) (Scheme 6). Starting from the promising results exhibited in the nitroaldol reaction, the Mets7/Cu(I) reactivity was further evaluated in the Michael addition reaction of nitromethane to a series of homocycle and heterocycle chalcones [80].

**Figure 4:**
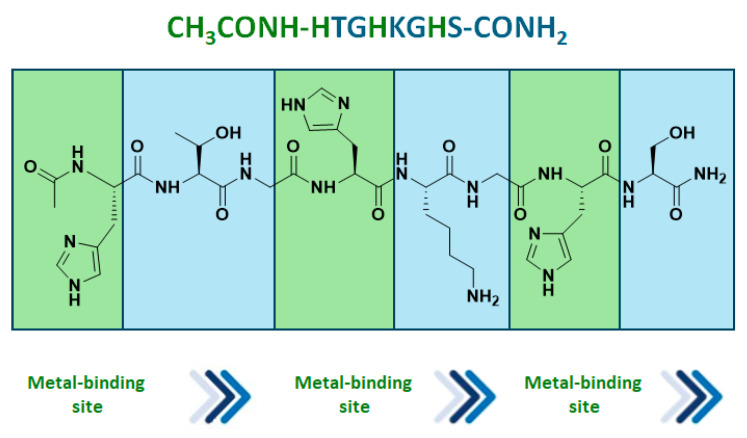

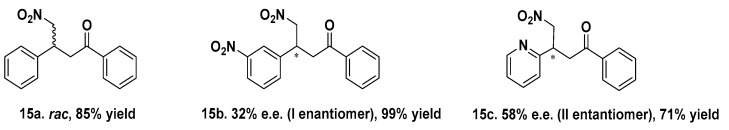
His7 peptide sequence and selected Michael reaction addition products obtained by His7/Cu(II) catalysts [80].

Mets7 reactivity as a ligand in copper MPs was further expanded by replacing the soft base Met residues with the harder base histidine in the corresponding His7 analogue (AcHTGHKGHS), thus allowing even for coordination to both Cu(I) and the borderline acid Cu(II). As a consequence of this simple structural change, the His7/Cu(II) complex was characterized by a square planar coordination geometry, which is thermodynamically more stable and inert to oxidation, thus avoiding the use of inert atmosphere conditions necessarily used for the preparation of Cu(I) hybrid systems. The conformational behavior of peptides and their complexes was investigated in water by CD experiments confirming a conformational induction of His7 to a β-turn-like structure more evident upon coordination to Cu(II) than Cu(I) and characterized by a strong negative value at 190 nm and a positive Cotton effect at 205 nm. The catalytic results showed His7/Cu(II) revealed to be the best catalyst in terms of conversion (up to 99%) when differently substituted chalcones were employed as substrates and with e.e. spanning from 32% for the *m*-NO_2_ **15b** to the 58% obtained with 2-pyridinyl-enone **15c** (Figure 4). Conversely, a significant decrease in reactivity was recorded for both Mets7/Cu(I) and His7/Cu(I) hybrid systems, probably due to the less conformational stability. 

**Figure 5:**
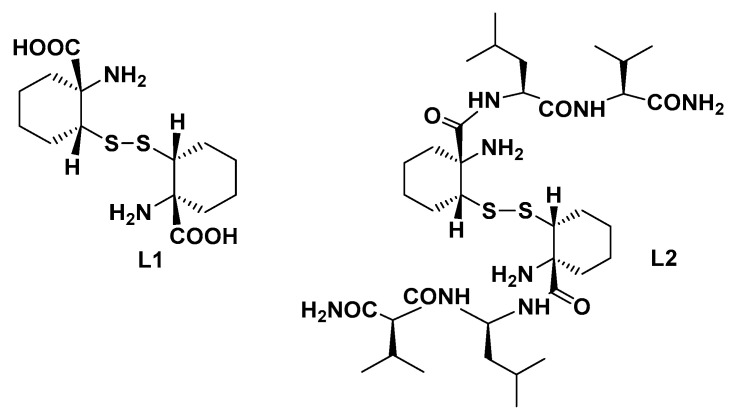
Structure of the constrained Cys derivatives **L1** and **L2** used for the preparation of Cu(II)-based MPs.

The use of ultrashort peptides containing unnatural Aas is indeed a valid approach for retaining specificity, but it does avoid peptide complexity both in the synthesis and conformational stability [81,82]. Our research group has recently reported the synthesis of a constrained cysteine analogue (**L1**, Figure 5) characterized by a tetrasubstituted Cα in which the H is substituted by an alkyl or an aryl group and thus, in principle, is able induce secondary structures when inserted in an ultra-short peptide [83]. This unnatural Aa was successively employed as a skeleton for the preparation of a short peptide by introducing the dipeptide Leu-Val at both of its C-termini (**L2**, Figure 5). Leu-Val was rationally selected considering its propensity to maintain an extended conformation due to electronic repulsions but also taking into consideration its ability to fold in a defined secondary structure in the presence of a structuring Aa such as the rigid cysteine analogue [84]. NOESY-NMR, FTIR, and CD analyses, supported by rMD (restrained Molecular Dynamics) simulations, confirmed for the peptide and its analogues the ability of these short peptides to assume a conformational stable turn-like structure. 

Upon Cu(II) coordination, the resulting MPs proved to be efficient catalysts in the asymmetric Michael condensation reaction of nitromethane with a series of substituted chalcones and a valuable synthetic way to obtain biologically active γ-aminobutyric acids with potential GABAergic modulation activity. In particular, the free cysteine scaffold (**L1**) proved a more versatile ligand than **L2**, providing satisfactory quantitative reaction yields with chalcones carrying an electron withdrawing group on the benzoyl moiety and enantioselectivity up to 33% e.e. for the *m*-CF_3_-substituted chalcone. Conversely, catalyst L2-Cu(II) proved a less reactive catalyst as a general trend, although when applied to 2-pyridinyl-enone, the reaction led to the addition product in striking 60% e.e. under solvent-free conditions. 

### 2.3. New Hybrid Catalytic Approaches in Friedel–Crafts Alkylation and Sonogashira Cross-Coupling

The Friedel–Crafts reaction could be defined as an organic chemistry synthetic approach providing the electrophilic substitution of aromatic rings and frequently turning out as the key step in the total synthesis of complex bioactive naturally occurring compounds [85,86]. 

Among the privileged scaffolds capitalized in the preparation of ArMs by different anchoring strategies, the human Steroid Carrier Protein (SCP-2L) is one of the most versatile [87,88]. Possessing a hydrophobic cavity suitable for hosting different metal cofactors, it has been largely exploited as a scaffold by Jarvis and Kamer’s group in the development of a Rh-based artificial catalytic system for regioselective hydroformylation [89] or in the selective oxidative degradation of lignin [90] and recently as a photoenzyme in the selective oxidation of organic sulfides [91]. Its versatility confirmed the idea that even proteins endowed with a carrier function could serve as a suitable scaffold for the synthesis of ArMs. 

Analogously to the privileged scaffolds in the field of ArMs, some transition metal ligands could be considered privileged as well in homogenous catalysis. Bipyridine can boast this privilege for sure, being used as a ligand for many transition metal complexes endowed with both catalytic and biological properties [92]. Not only can its bromomethyl derivative be introduced into proteins via bioconjugation selectively at a cysteine residue, but also it can be directly expressed in the protein-of-interest in *E. coli* systems through the Amber Codon Suppression technique [93]. 

**Figure 6:**
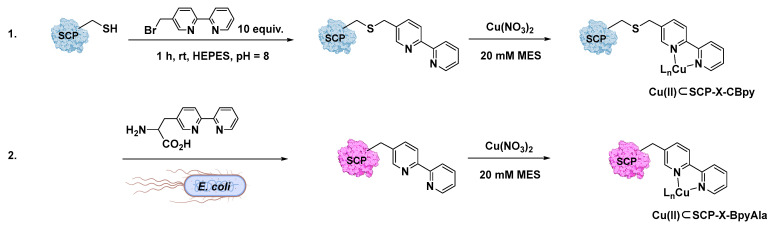
Different approaches for the preparation of Cu(II)⊂SCP ArMs. (**1**) In vitro bioconjugation of the Cys residue in the SCP with 5-bromomethyl-2,2-bipyridine before the addition of Cu(NO_3_)_2_ salt; (**2**) in vivo incorporation of the unnatural BpyAla. Reproduced from Ref. [94] with permission from the Royal Society of Chemistry.

Bipyridine (Bpy) was thus introduced into the protein scaffold through the bioconjugation of the cysteine residues with 10 equiv. of 5-bromomethyl-2,2-bipyridine in HEPES buffer at pH 8.0 [94]. According to this procedure (Figure 6), three different steroid carrier proteins were prepared, each containing a unique cysteine at position Q111C, A100C, or V83C. Conversely, genetic code expansion using stop codon suppression relies on the introduction of Bpy in the form of the unnatural amino acid BpyAla along with the protein expression. ESI-MS was used in both cases to confirm the successful introduction of the Bpy or as BpyAla within the host protein. The corresponding Cu(II)-ArMs were then synthesized by reaction with Cu(NO_3_)_2_, as demonstrated by UV–Vis and ICP-MS analyses showing a 1:1 binding stoichiometry for copper to the bipyridine ligand. X-ray crystallography structures were even realized for Cu(II) ⊂ SCP-Q111CBpy and Cu(II) ⊂ SCP-Q111BpyAla in order to obtain structural details of the catalytic moiety and insights about the environmental effects provided by the nearest Aas residues around the metal center. For Cu(II) ⊂ SCP-Q111CBpy, X-ray structures highlighted the presence of the Bpy ligand sandwiched between the amide sidechain atoms of Q108 and Q90 responsible for stabilizing aromatic π–amino electrostatic interactions with the Cu(II) ion, lying 2 Å from the two N atoms of the pyridine ring, and thus adopting an octahedral coordination geometry. 

In the case of Cu ⊂ SCP-Q111BpyAla, BpyAla stuck into a hydrophobic pocket formed by the turn between G85 and P89 residues on the surface of the host protein, which might contribute to stabilizing the metal cofactor by aromatic–π electrostatic interactions and make the catalytic pocket more rigid. 

**Scheme 7:**
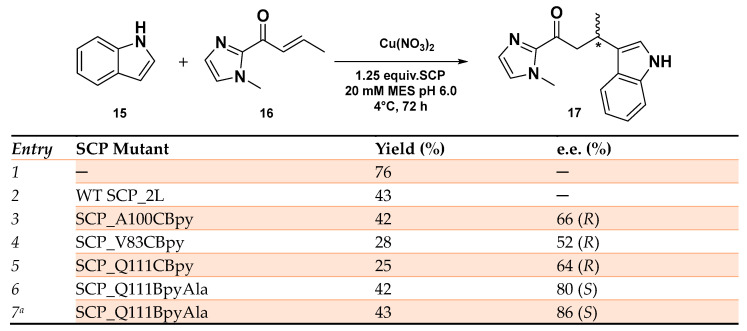
**Reaction conditions:** reactions were carried out using 9 mol% of Cu(NO_3_)_2_ (90 μM), SCP mutant (112.5 μM), 1 mM of **15** and 2.5 mM of **16** in MES buffer (20 mM, 150 mM NaCl, pH 6.0) for 3 days at 4 °C. [a] reaction was conducted in MES buffer 20 mM, 150 mM NaCl, pH = 5.0 [94].

The SCP-based ArMs were then evaluated as catalysts in the Friedel–Crafts alkylation reaction of 5-methoxy-1H-indole **16** with 1-(1-methyl-1H-imidazol-2-yl) but-2-en-1-one 17 in MES buffer at pH 6.0. Among the synthesized Cu-based hybrid systems, the ones carrying the BpyAla resulted in being more reactive with product yields in the range of 42–45%. Cu(II) ⊂ SCP-Q111CBpyAla showed the highest enantioselectivity, affording the product in 80% e.e. in favor of the (*S*)-enantiomer. Crystallographic data revealed that, in this case, the BpyAla residue was set in the middle of α-helix 5 at the center of the hydrophobic pocket, whereas by moving the metal cofactor towards the end of the catalytic site, as in the Cu(II) ⊂ SCP-A100CBpyAla, the enantioselectivity was reversed, showing a little preference for the (*R*)-enantiomer (52% e.e. (*R*), 45% yield). Conversely, with all the three CBpy mutants, the product could be obtained only in (*R*) configuration but in 66% e.e. with Cu(II) ⊂ SCP_A100CBpy system, probably due to a greater flexibility of the -CH_2_-S linker around the catalytic site (Scheme 7). The enantioselectivity outcome was rationalized by computational studies confirming that a different anchoring of the catalytic cofactor led to a stabilization of indole substrate on different sides on the but-2-en-1-one **15**, according to the transition state energy barriers calculated for the pro-(*R*) and pro-(*S*) mutants’ models. While SCP-Q111CBpyAla would favor the pro-(*S*) over pro-(*R*) product, the opposite situation was established with SCP-Q111CBpy. The higher differences in energy calculated for the SCP-Q111CBpyAla TS intermediates perfectly matches the higher enantioselectivity experimentally achieved with Cu(II) ⊂ SCP-Q111CBpyAla. The further evolution of Cu(II) ⊂ SCP-Q111CBpyAla by single residue mutagenesis confirmed the Q111 mutant as the most promising in the SCP-based ArMs affording the Friedel–Crafts product with a slightly enhanced 86% e.e. (*S*) when the reaction took place in MES buffer at pH 5.0. 

**Scheme 8:**
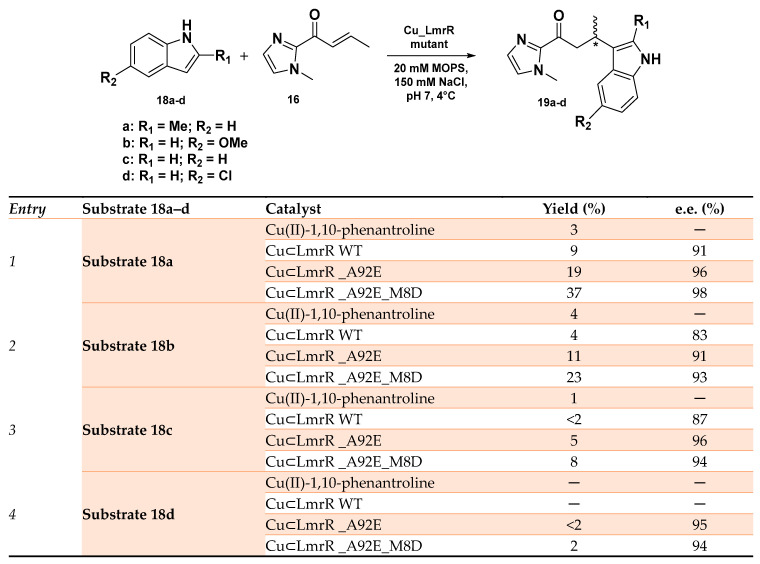
**Reaction conditions:** reactions were carried out using 12 mM LmrR mutant, 9 mM Cu(II)-Phen, 1 mM **18a**–**d** and **16** in 20 mM MOPS, 150 mM NaCl, pH 7.0 at 4 °C [99].

LmrR (Lactococcal multidrug resistance Regulator) represents another privileged scaffold employed in the preparation of ArMs for enantioselective Friedel–Crafts reactions [95,96,97]. This transcriptional factor exists as a homodimer with a large hydrophobic pocket at the dimer interface and is able to promiscuously host different planar small molecules. A Cu(II)-1,10-phenantroline cofactor was inserted within this cavity in proximity to two tryptophan residues W96 and W’96, one for each monomer, both critical in stabilizing the metal cofactor (K_d_ of 2.6 μM). In the LmrR_LM_W96A, which contains an alanine residue in place of the tryptophan in the hydrophobic pocket, the binding of the Cu(II) complex resulted in being extremely weaker, i.e., with a K_d_ of 45 μM. The so ArMs were then applied to the enantioselective Friedel−Crafts alkylation of 5-methoxy-1H-indole with 1-(1-methyl-1H-imidazol-2-yl) but-2-en-1-one, displaying enantioselectivity up to 94% e.e. (*R*) with quantitative conversion [98]. Among the different mutants obtained by mutagenesis experiments, LmrR_A92E showed an increased selectivity for Friedel–Crafts alkylation if compared to other competitive reactions due to a different arrangement of the indoles of W96 and W’96 residues, resulting in an expanded hydrophobic pocket able to host the Cu(II)-1,10-phenantroline complex deeper inside the pore. The same group recently reported the spontaneous self-assembly of LmrR-based ArMs in the cytoplasm of *Escherichia coli* and their use as whole cells as catalysts in the same reaction [99]. After having demonstrated the existence of a reliable correlation between the activity and selectivity reported for the isolated ArMs and the one assembled in *E. coli* whole cells, directed evolution was applied in order to optimize the reaction outcome. LmrR_A92E was selected as the starting point for such a series of experiments considering its higher reactivity among the LmrR mutants, and, through an Alanine Scan study, a series of influencing residues in the hydrophobic pocket were identified for producing a library of newly expressed mutants in cells. Cu ⊂ LmrR _A92E_M8D resulted in being the most promising in the selective Friedel–Crafts alkylation reaction of differently substituted indoles **18a**–**d** (Scheme 8) with excellent enantiomeric excess up to 98% e.e. along with a modest product yield (substrate **18a**), overcoming in all cases the WT-based hybrid system and demonstrating the beneficial effect of the protein scaffold optimization. However, this is a very sophisticated example of how it was possible to modulate cell metabolism by abiological catalysis [99]. 

In the search for a more sustainable catalysis, SACs (Single-Atom Catalysts) have attracted extensive attention due to the high catalytic activity achievable, maximum atom exploitation, and reduced metal use [100,101]. The SAC approach provides single metal atoms anchored on suitable supports such as graphene or different metal oxides, although these hybrid catalysts should be distinguished from nanoparticles considering the absence of any metal–metal bond in the former case [102]. The non-innocent supports in such a type of catalyst display the same role as the ligands in organometallic catalysts, thus enabling charge transfer to the metal and providing a structurally defined environment. Being at the interface between homogeneous and heterogenous catalysis, this emerging field offers interesting opportunities for mimicking metalloenzymes [103]. Moreover, the single metal atoms are usually positive charged, thus introducing electronic and geometric properties that are able to significantly impact the interaction with the substrates and the reaction intermediates, taking account of the higher reactivity and selectivity observed with SACs [100,104]. 

**Scheme 9:**
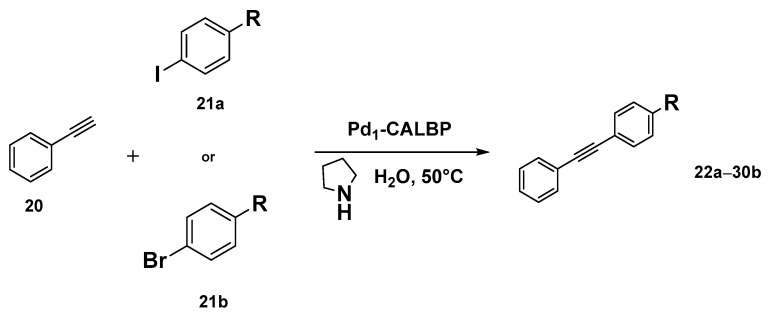

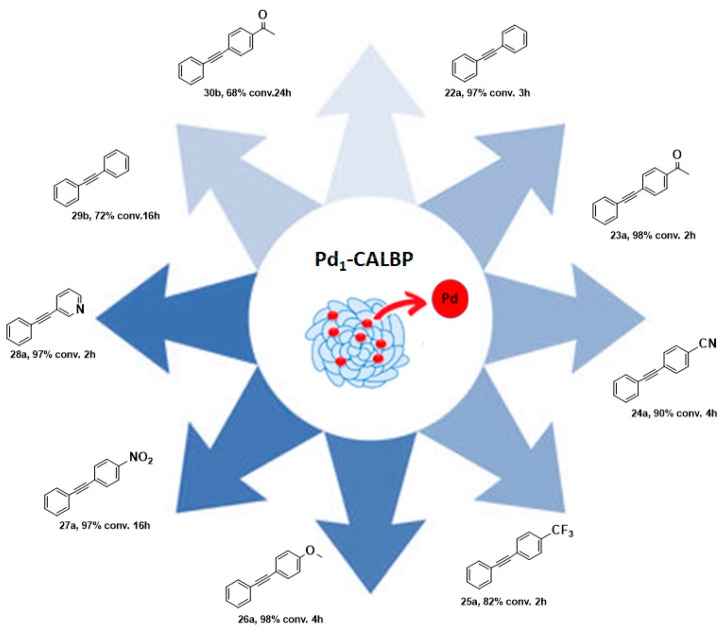
**Reaction conditions:** reactions were carried out using Pd_1_-CALBP catalyst 0.014 mmol, 1.2 mmol **20**, aryl halide **21a** or **21b** 1.0 mmol, pyrrolidine 450 µL, 50 °C, 2–16 h [105].

Taking inspiration from the SAC world, Ge et al. recently reported the preparation of an enzyme–metal–single-atom Pd-anchored lipase (Pd_1_-CALBP) as an efficient catalyst in the Sonogashira C─C bond formation reaction in water [105]. The hybrid catalyst was obtained by anchoring the single Pd atoms on the lipase–Pluronic adduct by using a photochemical method. AC-STEM and EXAFS analyses along with computational studies were used to confirm the uniform distribution of Pd atoms on the lipase and the absence of Pd-Pd bonds. DFT calculations and MD simulations, indeed, suggested Asp223 as the most probable linking residue of Pd atoms to the enzyme active sites. The Pd_1_-CALBP was then applied to the Sonogashira cross-coupling reaction of iodobenzene **21a** with phenylacetylene **20** in aqueous solution at 50 °C in the presence of pyrrolidine, affording the desired product with TOF resulting in being 2.3-fold, 3.5-fold, and 2.2-fold higher than Pd/C, PdNPs ⊂ CALBP, and the metal precursor PdCl_2_, respectively. The presence of Pluronic copolymer on lipase surface, indeed, enhances the Sonogashira coupling reaction in water, preventing enzyme aggregation, avoiding its interfacial denaturation, and enabling the use of reactants typically insoluble in an aqueous medium [106,107]. Furthermore, Pd_1_-CALBP showcased reusability, maintaining more than 93% of activity even after ten cycles. To highlight the reactivity of the system and its versatility, the Sonogashira reaction was extended to different aryl halides. Excellent yields up to 98% were obtained with those bearing electron-withdrawing substituents such as -CN, -CF_3_, and -NO_2_ in 2–16 h (Scheme 9). Analogous reactivity was observed even with heteroaromatic aryl halides leading to a quantitative formation of the product within 2 h. The catalyst proved its surprising activity even with aryl bromides **21b**, affording 72% and 68% yields in the reactions using bromobenzene and 4′-bromoacetophenone as the substrates, respectively.

The authors moreover applied the Pd_1_-CALBP catalyst in a one-pot reaction to produce (*R*)-1-(4-biphenyl) ethanol from racemic 1-(4-bromophenyl) ethyl acetate by combining lipase-catalyzed stereo-selective hydrolysis and a Pd-Suzuki cross coupling, reaching 96% of selectivity and 99.7% e.e. in the synthesis of (*R*)-1-(4-biphenyl) ethanol. It is worth noting that the reaction could be performed in aqueous solution at 30 °C at a substrate concentration of 100 mmol/L in a 30-fold faster rate than the commercial Pd/C and CALBP [108].

### 2.4. ArMs and MPs in Cyclopropanation Reactions

Cyclopropanes are considered to be a privileged structure expressed in both natural products and in synthetic bioactive compounds [109]. The peculiar steric and electronic features of the constrained cyclopropane ring make it a valuable motif in the medicinal chemistry field that is able to boost selectivity, binding properties with the designed target, and bioavailability but also decrease metabolic instability, lipophilicity, and off-target interactions [110,111]. Its introduction in the architecture of bioactive molecules can be traced back to 1960s. and it is exemplified in monoamine oxidase inactivators such as tranylcypromine [112] and in opioid antagonists’ naltrexone [113]. 

**Scheme 10:**
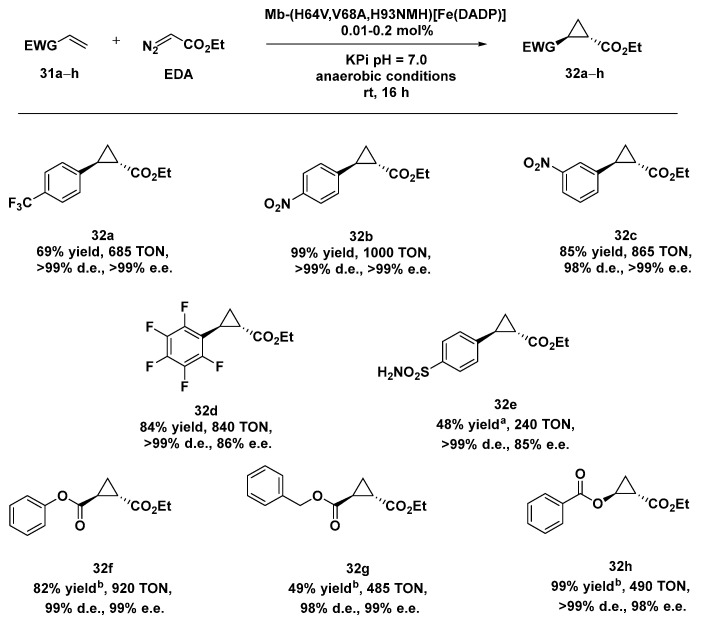
**Reaction conditions:** reactions were carried out using 10 mM alkene **31a**–**h**, 20 mM EDA, 10 µM Mb variant in 50 mM KPi buffer pH = 7.0, rt, 16 h, anaerobic conditions. [a] 20 mM alkene **30e**, 10 mM EDA, 20 µM Mb variant; [b] 2.5 mM alkene **31f**–**h**, 2.5 mM EDA, 5 µM Mb variant. [117].

Enginreed myglobins (Mbs) have been extensively applied to the cyclopropanation of aryl-substituted olefins and aliphatic olefins [114,115], but the same systems resulted in being ineffective in the case of electron-deficient alkenes due to the electrophilic character of the carbenes typically involved in these reactions. In order to overcome these limitations, both the heme cofactor and the proximal ligand were modified in order to enhance the electrophilic reactivity of the heme-carbene intermediate. Starting from the Mb(H64V,V68A) variant, previously identified for its excellent stereoselectivity in the cyclopropanation of vinylarenes with EDA [116], the native heme in Mb(H64V,V68A) was substituted with an electron-deficient heme analogue, i.e., Fe(II)-2,4-diacetyl deuteroporphyrin IX (Fe(DADP)), with the effect to increase the Fe^3+/^Fe^2+^ reduction potential of Mb. A second variant was obtained by introducing *N*-methyl histidine (NMH) at the axial position of the Fe(DADP) cofactor in place of the natural histidine His 93, envisioning to further increase the redox properties of the system and thus synergistically modify the reactivity of such a hybrid carbene transferase [117]. The preliminary results obtained with both Mb variants, Mb-(H64V,V68A)[Fe(DADP)] and Mb-(H64V,V68A,H93NMH)[Fe(DADP)] in the cyclopropanation of aryl- and hetero-atom-substituted olefinsshowed an excellent yield, *trans* diastereoselectivity (98 to >99% d.e.), and (1*S*,2*S*)-enantioselectivity (98% to >99% e.e.) with electron-donating and electron-withdrawing groups on the aryl moiety. The higher enantioselectivity levels observed with the monohalogenated styrenes let the authors identify Mb-(H64V,V68A,H93NMH)[Fe(DADP)] as the best one to be applied in the cyclopropanation of electron-deficient olefins, under anaerobic conditions. Even in this case the desired products could be isolated in good-to-quantitative yields and had both excellent distereo- and enantioselectivity (Scheme 10). 

**Scheme 11:**
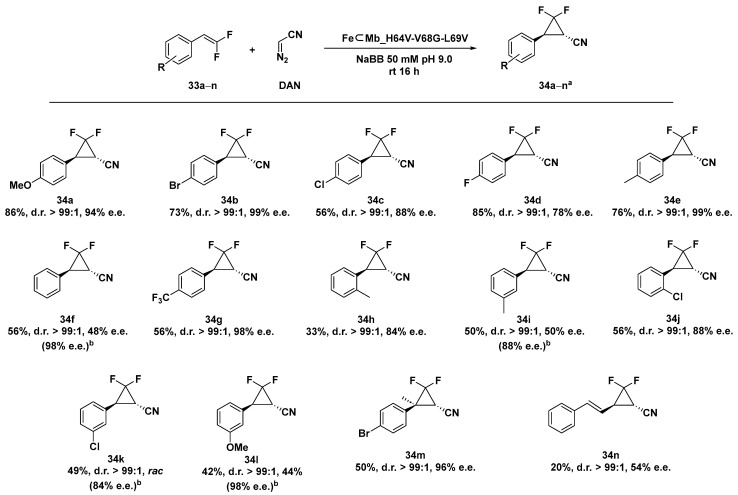
**Reaction conditions:** reactions were carried out using 5 mM olefin **33a**–**n**, 20 mM **DAN**, Fe ⊂ Mb_H64V-V68G-L69V expressing *E. coli* in 50 mM NaBB (sodium borate buffer) pH 9.0, rt 16 h, anaerobic conditions. [a] Yield as determined by fluorine NMR in presence of trifluorotoluene as internal standard. [b] e.e. obtained using Fe ⊂ Mb_H64A-V68G-L69V mutant [125].

Fluorinated cyclopropanes provide a class of cyclopropyl-containing pharmacophores particularly desired in drug discovery, taking advantage of the presence of both the rigid cyclopropane ring and the favorable properties of fluorine substituents [118,119] There are few synthetic methods reported in the literature for the preparation of enantioenriched fluorinated cyclopropanes including the kinetic resolution of racemic difluoro cyclopropyl esters [120], the asymmetric hydrogenation of fluorocyclopropenes [121], and the asymmetric cyclopropanation of fluorosubstituted allylic alcohols [122]. The myoglobin (Mb) mutant Fe ⊂ Mb_H64V-V68A has already been demonstrated to be an efficient catalyst in cyclopropanation reactions [117,123,124], with the exception of the synthesis of *gem*-difluoro cyclopropane products. By applying mutagenesis optimization experiments, the two Mb variants Fe ⊂ Mb_H64A-V68G and Fe ⊂ Mb_H64V-V68G proved to be more suitable as catalysts in the cyclopropanation of difluorinated styrene **32a**–**n** with diazoacetonitrile (**DAN**), leading to the desired *gem*-difluoro-cyclopropane with high diastereoselectivity (99:1 d.r.) and enantiomeric excess up to 99% e.e. for Fe ⊂ Mb_H64A-V68G and 97% for Fe ⊂ Mb_H64V-V68G in 16 h at rt [125]. Despite the excellent selectivity levels, the previous two ArMs were able to afford the product in very modest yields (10% and 14% for Fe ⊂ Mb_H64A-V68G and Fe ⊂ Mb_H64V-V68G, respectively). Significantly, the two single-site variants were not effective in the same reaction, highlighting that the two mutations were both necessary for expanding the reactivity of such a hybrid system to difluorinated olefins. In order to further improve the selectivity and the yield of the reaction, mutagenesis studies were extended to residue 69, proximal to the already mutated 68 position but far from the catalytically active iron cofactor. Ala or Val introduction in place of a Leu69 residue in the second sphere successfully allowed us to increase the yield up to a remarkable 86% with Fe ⊂ Mb_H64V-V68G-L69V. The catalytic system demonstrated a good tolerance towards both different electron- donating or electron-withdrawing groups on the difluorinated styrene providing the corresponding *gem*-difluoro-cyclopropanes in moderate-to-good yield (20–86%) and with excellent diastereoselectivity (up to > 99:1 d.r.) and enantioselectivity (up to > 98% e.e.) as shown in Scheme 11. It is interesting to note that the enantioselectivity levels across the *para*-substituted series of difluorinated styrene emerged to grow with the increasing size of the *para*-substituent: **34b** (-Br) and **34e** (-Me) 99% e.e. > **34a** (-OMe) 94% e.e. > **34c** (-Cl) 88% e.e. > **34d** (-F) 78% e.e. > **34f** (-H) 48% e.e. despite their different electronic effects. Moreover, the catalytic system demonstrated its reactivity even when applied to both *meta-* and *ortho*-substituted styrenes, although with a slight erosion of the reaction yield as a general trend along with a net decrease in the enantioselectivity of the *meta*-products. To overcome this limitation and by combining crystallographic with computational DFT analyses, a Val residue was selectively introduced at position 64 to better accommodate the *meta* substituent in the active site of the ArM. Thus, substrates **33f**, **33i**, **33k,** and **33l** were efficiently transformed in the corresponding cyclopropanated products with excellent enantioselectivity up to 98% e.e. by the customized mutant Fe ⊂ Mb_H64A-V68G-L69V. 

**Scheme 12:**
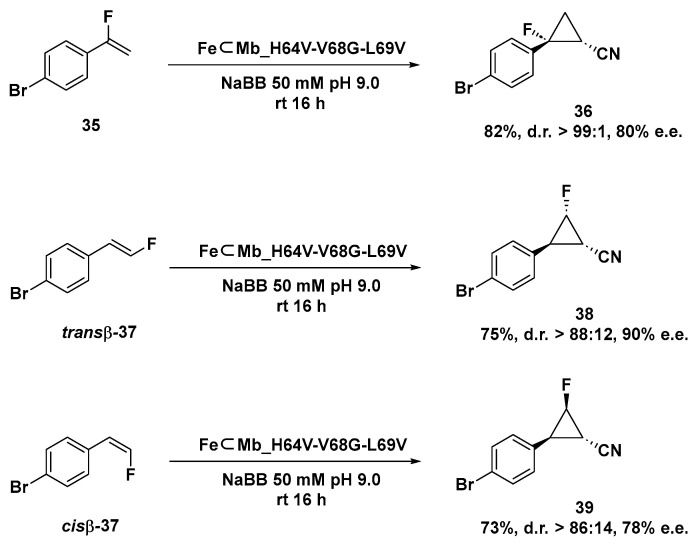
Cyclopropanation reaction of monofluorinated styrene substrates catalyzed by Fe ⊂ Mb_H64V-V68G-L69V mutant [125].

Gratifyingly, the Fe ⊂ Mb_H64V-V68G-L69V variant demonstrated its selective and versatile reactivity even when applied to the asymmetric cyclopropanation of both α-fluoro styrene **35** and *trans*-β or *cis*-β styrene as shown in Scheme 12. Finally, the authors showed the possibility to reverse the enantioselectivity of the artificial catalytic system by expressing the Fe ⊂ Mb_ L29V-F33A-H64T-V68L mutant, which is able to produce compound **34a** in 46% yield in excellent enantiopurity but in opposite configuration, highlighting the possibility to conduct this transformation with enantiodivergent selectivity.

Allene cyclopropanation still provides a challenging transformation [126], especially when a stereocontrol of both the configuration of the cyclopropyl (*R* vs. *S*) group and the geometry of the alkene (*E* vs. *Z*) are desired, with only few examples reported in the literature [127,128,129]. Indeed, allenes show diminished reactivity compared to alkenes toward cyclopropanation reactions due to a poor nucleophilicity as a result of the electron-deficient central *sp* carbon, with the terminal olefin more sterically favored in comparison to the internal olefin, which is conversely electronically favored. Furthermore, the two faces of the terminal alkene in 1,1-disubstituted allenes show a diastereotopic behavior affording to a mixture of *E*/*Z* products. Encouraged by the results obtained in the cyclopropanation of terpenoid-like substrates by the iridium-containing cytochrome hybrid system Ir(Me)_CYP119 [59], Iterative Saturation Mutagenesis (ISM) was exploited for unravelling those residues involved in determining both the reactivity and selectivity of the ArM [130]. A six-codon strategy was thus applied to produce a series of mutants endowed with different selectivity, involving eight sterically different residues present in the active site of the protein scaffold. A first round of evolution produced the Ir(Me)_CYP119-A209I mutant able to afford *E*(-)-alkylidene cyclopropane in a remarkable 93% e.e. as a single diastereomer. The introduction of two single mutations (A152G and G213L) reversed the enantioselectivity, leading to the isolation of the corresponding *E* (+)-product with 94% e.e., which could be further increased to 99% e.e. with an additional I205G mutation. Remarkably, the purified mutant allowed us to produce the *E* (+)-alkylidene cyclopropane on a 1 mmol scale in 69% yield, 97% e.e., 97:1:2 ratio of 40*E*/40*Z*/40MCP (*E*, *Z* and MCP = methylene cyclopropane, respectively), and in TON of 909. Indeed, X-ray crystallography studies confirmed the enantiopurity of the product and its (*R*) absolute configuration. 

**Scheme 13:**
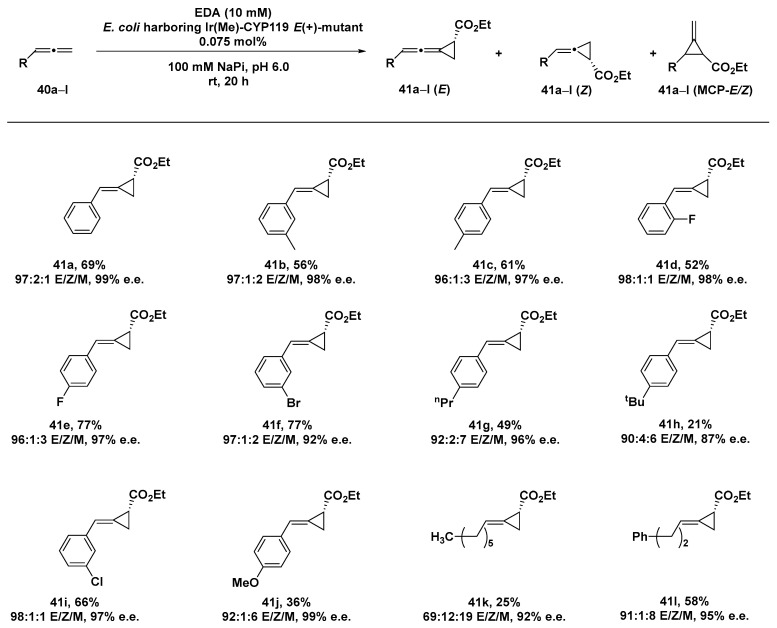
**Reaction conditions:** reactions were carried out using 0.075 mol% Ir(Me)-CYP119 *E*(+)-mutant, 0.5 mmol (5 mM) allene **40a**–**l**, 10 mmol **EDA**, 100 mM NaPi (sodium phosphate buffer) pH 6.0, rt, 20 h [130].

By rationally inserting mutations L205F/G213A/F153V in order to better accommodate the aromatic moiety of the allene substrate, even the *Z* (-)-isomer, never achieved before by small-molecule catalysts, could be isolated as the major one (28:72 *E*/*Z*) with an excellent 94% e.e. The substrate scope of the reaction was investigated in terms of different steric hindrances exerted by the substituents on the phenylallene substrates **40a**–**l**, showing a good tolerance of both small fluoro-, methyl-, methoxy-, and chloro-substituents and larger *n*-propyl- and bromo-groups. In the presence of the bulkier *t*-butyl group **41h**, a sensible decrease in enantioselection could be detected, even more significant if compared to the more flexible aliphatic *n*-hexyl- and phenethyl derivatives **41k** and **41l**, respectively. For these phenylallene substrates, an increase in the MCP isomer was conversely produced, although retaining the excellent enantioselectivity unaltered (Scheme 13).

All these data were supported by Density Functional Theory (DFT) calculations and Molecular Dynamics (MD) simulations, showing the effect exerted by the inserted mutations in stabilizing different orientations of the Ir-carbene intermediate involved in the reaction pathway and found responsible for the different enantio- and chemoselectivity outcome. Hydrophobic interactions of the terminal alkene of the allene substrate with W155, V151, A152, and V353 residues proved to directly impact modulating the pro-(*E*) or pro-(*Z*) binding mode with the Ir-carbene intermediate [59]. 

As already mentioned, MPs in addition to their more affordable synthesis, if compared to the expression of whole proteins, are well-suited scaffolds for preparing supramolecular structures endowed with catalytic properties thanks to their functional groups that are able to form hydrogen bonding, aromatic stacking, and van der Waals interactions [131]. 

**Scheme 14:**
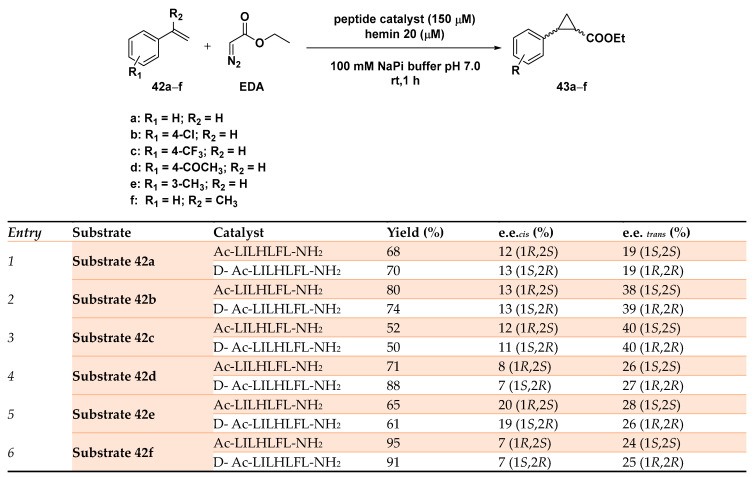
Screening of the substrate scope of the cyclopropanation reaction catalyzed by the MP-based hybrid system. **Reaction conditions**: reactions were carried out using 150 µM peptide with 20 µM hemin, 8mM styrene substrate **42a**–**e**, 24 mM **EDA**, 10 mM dithionite, 100 mM NaPi (sodium phosphate buffer) pH 7.0, rt, 1 h [132].

An original approach in the asymmetric cyclopropanation reported the use of a series of seven-residue peptides able to form hemin-binding assemblies to support the cyclopropanation reaction of 4-(trifluoromethyl) styrene with **EDA** (ethyl diazoacetate) [132]. Starting from a small library of peptides capable of self-assembling in amyloids and after having tested their ability to coordinate hemin via red shift in the Soret band, peptide Ac-LHLHLFL-NH_2_ was identified as the most promising of the series. The β-sheet assemblies resulted in being able to promote the diastereo- and enantiospecific cyclopropanation of 4-(trifluoromethyl) styrene with **EDA**, although in modest yield (up to 48%) and selectivity (72% d.e., *rac*). Considering that all the screened peptides proved their ability to both self-assemble and to coordinate to the metal cofactor, catalytic data proved Ac-LILHLFL-NH_2_ as the peptide able to afford the best results in terms of enantioselectivity (52% yield, 68% d.e., 40% e.e. *trans* 1*S*,2*S*). Computational studies showed that only the histidine residue in fourth position was not only involved in iron coordination within the hemin scaffold but also in determining the overall reactivity of the artificial system. In fact, its substitution with the unnatural Aa 3-Me histidine, often employed to boost the catalytic performance of engineered myoglobins [117], was not able to induce any enantioselectivity in the cyclopropanation reaction, showing the importance of hydrogen bonding arising from the histidine side chain for hemin coordination in a catalytically effective conformation. A second round of sequence changes was then realized in which the effect of different residues in position 2 was evaluated, as well as peptides incapable of forming supramolecular structures but preserving the hemin-binding ability; this included the more hydrophobic and methylated Ac-LHLH(L-NMe)FL-NH_2_ and its hydrophilic counterpart Ac-AHAHAFA-NH_2_, respectively. The complete lack of entantioselectivity afforded in the cyclopropanation reaction by these peptides in the presence of the iron cofactor unambiguously demonstrated the necessity of proper self-assembly in higher order supramolecular structures for obtaining enantioselective catalysts. Starting from these results, Ac-LILHLFL-NH_2_ was established as the peptide of choice for conducting the cycloprapanation reaction on different styrene substrates **42a**–**f**. The artificial system showcased a good tolerance with both electron-donating and electron-poor substrates resulting in being enantioselective in all cases, with a little preference for the *trans*-1*S*,2*S* products (**43a**–**f**) as depicted in Scheme 14. 

The best result was obtained indeed in the cyclopropanation of **42c**, leading to the product with a 40% e.e. in only 1 h in phosphate buffer, pH 7.0. For the sake of comparison, hemin used alone did promote the reaction only with 70% d.e. and without enantioselectivity. Interestingly, the use of the corresponding D-amino acid-based peptides showed a complete reversal of the enantiopreference, a property impossible to realize with native enzymes that limit their applicability in such types of catalyzed reactions. Although modest enantioselective levels were achieved, these hybrid catalytic systems showed great potential for customization stemming from the assemblies of short peptides in building up a well-defined second coordination sphere around the metal cofactor and affording catalysts’ efficiencies comparable to some engineered proteins [133].

## 3. Conclusions and Future Directions

Since their introduction by Wilson and Whitesides in 1978, Artificial Metalloenzymes (ArMs) have gained attention in the field of catalysis, offering a unique platform for abiotic reaction development. With the idea to combine the best aspects of both homogeneous and enzymatic catalysis, unnatural metal cofactors were selectively introduced into well-defined coordination spheres, thus greatly expanding the naturally evolved reaction repertoire. Although a large-scale application still remains an unmet goal till now because of the challenges associated with their production and optimization, their water compatibility has attracted remarkable attention with the idea to develop a more sustainable way to perform catalysis. On the other hand, the extensive knowledge acquired regarding peptide synthesis, along with the possibility to expand their structural composition by the introduction of unnatural amino acid residues, has introduced the possibility to exploit peptides as precise replicas of protein fragments. Inspired by the synergistic cooperation between metal ions and proteins in natural metalloenzymes, Metallo Peptides (MPs) represent an innovative approach for mimicking the activities of metalloproteins by identifying the least number of residue interactions necessary to maintain or to reverse completely the catalytic reactivity of the metal center. Headways in both rational protein engineering and the directed evolution coupled with progress in in silico studies have made ArMs and MPs a new versatile tool in organic synthesis. 

Among the plethora of organic transformations catalyzed by these two classes of hybrid systems, this review delves into the recent advances in some important reactions to asymmetrically construct the carbon framework of biologically relevant molecules. 

Finally, the advancements in both organometallic catalysis and emerging technologies, including genome mining and computational design, have enabled us to broaden the scope of reactions even into complex biological media. Recent examples of in vivo catalysis in the literature have demonstrated that catalytically active ArMs can effectively self-assemble within cells. These studies represent a significant milestone in the field, showcasing that hybrid metabolism is not only feasible but also marks a clear step forward in developing advanced drug therapy.

## References

[1] Isahak W.N.R.W., Al-Amiery A. (2024). Catalysts driving efficiency and innovation in thermal reactions: A comprehensive review. Green. Technol. Sustain..

[2] James C.C., de Bruin B., Reek J.N.H. (2023). Transition Metal Catalysis in Living Cells: Progress, Challenges, and Novel Supramolecular Solutions. Angew. Chem. Int. Ed..

[3] Docherty J.H., Lister T.M., McArthur G., Findlay M.T., Domingo-Legarda P., Kenyon J., Choudhary S., Larrosa I. (2023). Transition-Metal-Catalyzed C–H Bond Activation for the Formation of C–C Bonds in Complex Molecules. Chem. Rev..

[4] Yang H., Yu H., Stolarzewicz I.A., Tang W. (2023). Enantioselective Transformations in the Synthesis of Therapeutic Agents. Chem. Rev..

[5] Yang X., Jiang S., Jin Z., Li T. (2024). Application of Asymmetric Catalysis in Chiral Pesticide Active Molecule Synthesis. J. Agric. Food Chem..

[6] Lakhani P., Modi C.K. (2023). Shaping enantiochemistry: Recent advances in enantioselective reactions via heterogeneous chiral catalysis. Mol. Catal..

[7] Steinlandt P.S., Zhang L., Meggers E. (2023). Metal stereogenicity in asymmetric transition metal catalysis. Chem. Rev..

[8] Marianov A.N., Jiang Y., Baiker A., Huang J. (2023). Homogeneous and heterogeneous strategies of enantioselective hydrogenation: Critical evaluation and future prospects. Chem. Catal..

[9] Nuzhdin A.L., Bukhtiyarova M.V., Bukhtiyarova G.A. (2024). Organic synthesis in flow mode by selective liquid-phase hydrogenation over heterogeneous non-noble metal catalysts. Org. Biomol. Chem..

[10] Gupta A., Gupta R., Arora G., Yadav P., Sharma R.K. (2023). Heterogeneous Catalysis under Continuous Flow Conditions. Curr. Org. Chem..

[11] González-Granda S., Albarrán-Velo J., Lavandera I., Gotor-Fernández V. (2023). Expanding the Synthetic Toolbox through Metal–Enzyme Cascade Reactions. Chem. Rev..

[12] Das S., Pradhan T.K., Samanta R. (2024). Recent Progress on Transition Metal Catalyzed Macrocyclizations Based on C−H Bond Activation at Heterocyclic Scaffolds. Chem.-Asian J..

[13] Song F., Wang B., Shi Z.-J. (2023). Transition-Metal-Catalyzed C–C Bond Formation from C–C Activation. Acc. Chem. Res..

[14] Zhang X. (2024). Cyclization Strategies in Carbonyl–Olefin Metathesis: An Up-to-Date Review. Molecules.

[15] Jose J., Diana E.J., Mathew T.V., Rahimpour M.R., Makarem M.A., Roostaie T., Meshksar M. (2025). Chapter 9—Olefin metathesis homogeneous catalysts. Homogeneous Hydrogenation and Metathesis Reactions.

[16] Hoveyda A.H., Qin C., Sui X.Z., Liu Q., Li X., Nikbakht A. (2023). Taking Olefin Metathesis to the Limit: Stereocontrolled Synthesis of Trisubstituted Alkenes. Acc. Chem. Res..

[17] Fu D., Wang Z., Liu Q., Prettyman S.J., Solan G.A., Sun W.-H. (2024). Organometallic Mn(I) Complexes in Asymmetric Catalytic (Transfer) Hydrogenation and Related Transformations. ChemCatChem.

[18] Ansari M.F., Anshika, Sortais J.-B., Elangovan S. (2024). Transition-Metal-Catalysed Transfer Hydrogenation Reactions with Glycerol and Carbohydrates as Hydrogen Donors. Eur. J. Org. Chem..

[19] Lückemeier L., Pierau M., Glorius F. (2023). Asymmetric arene hydrogenation: Towards sustainability and application. Chem. Soc. Rev..

[20] Facchetti G., Cesarotti E., Pellizzoni M., Zerla D., Rimoldi I. (2012). “In situ” Activation of Racemic RuII Complexes: Separation of trans and cis Species and Their Application in Asymmetric Reduction. Eur. J. Inorg. Chem..

[21] Facchetti G., Fusè M., Pecoraro T., Nava D., Rimoldi I. (2021). New sp3 diphosphine-based rhodium catalysts for the asymmetric conjugate addition of aryl boronic acids to 3-azaarylpropenones. New J. Chem..

[22] Islam M.T., Bitu N.A., Chaki B.M., Hossain M.J., Asraf M.A., Hossen M.F., Kudrat-E-Zahan M., Latif M.A. (2024). Water-soluble Schiff base ligands and metal complexes: An overview considering green solvent. RSC Adv..

[23] Baricelli P.J., Pereira J.C., Rosales M. (2025). Aqueous-biphasic catalysis: A technological alternative for the use of organometallic complexes in hydrogenation and hydroformylation reactions with possible industrial application. Catal. Today.

[24] Yan Q., Wu X., Jiang H., Wang H., Xu F., Li H., Zhang H., Yang S. (2024). Transition metals-catalyzed amination of biomass feedstocks for sustainable construction of N-heterocycles. Coord. Chem. Rev..

[25] Habib U., Ahmad F., Awais M., Naz N., Aslam M., Urooj M., Moqeem A., Tahseen H., Waqar A., Sajid M. (2023). Sustainable Catalysis: Navigating Challenges and Embracing Opportunities for a Greener Future. J. Chem. Environ..

[26] Wilson M.E., Whitesides G.M. (1978). Conversion of a protein to a homogeneous asymmetric hydrogenation catalyst by site-specific modification with a diphosphinerhodium (I) moiety. J. Am. Chem. Soc..

[27] Morita I., Ward T.R. (2024). Recent advances in the design and optimization of artificial metalloenzymes. Curr. Opin. Chem. Biol..

[28] LIU X.-y., HUANG C.-q., JIN X.-r., LUO Y.-z. (2023). Research Progress of Artificial Metalloenzymes. China Biotechnol..

[29] Jeong W.J., Lee J., Eom H., Song W.J. (2023). A Specific Guide for Metalloenzyme Designers: Introduction and Evolution of Metal-Coordination Spheres Embedded in Protein Environments. Acc. Chem. Res..

[30] Wittwer M., Markel U., Schiffels J., Okuda J., Sauer D.F., Schwaneberg U. (2021). Engineering and emerging applications of artificial metalloenzymes with whole cells. Nat. Catal..

[31] Lee J., Yang M., Song W.J. (2023). The expanded landscape of metalloproteins by genetic incorporation of noncanonical amino acids. Bull. Korean Chem. Soc..

[32] Yu Y., Hu C., Xia L., Wang J. (2018). Artificial Metalloenzyme Design with Unnatural Amino Acids and Non-Native Cofactors. ACS Catal..

[33] Miller A.H., Blagova E.V., Large B., Booth R.L., Wilson K.S., Duhme-Klair A.-K. (2024). Catch-and-Release: The Assembly, Immobilization, and Recycling of Redox-Reversible Artificial Metalloenzymes. ACS Catal..

[34] Facchetti G., Bucci R., Fusè M., Erba E., Gandolfi R., Pellegrino S., Rimoldi I. (2021). Alternative Strategy to Obtain Artificial Imine Reductase by Exploiting Vancomycin/D-Ala-D-Ala Interactions with an Iridium Metal Complex. Inorg. Chem..

[35] Duran-Meza E., Araya-Secchi R., Romero-Hasler P., Soto-Bustamante E.A., Castro-Fernandez V., Castillo-Caceres C., Monasterio O., Diaz-Espinoza R. (2024). Metal Ions Can Modulate the Self-Assembly and Activity of Catalytic Peptide Amyloids. Langmuir.

[36] Leone L., De Fenza M., Esposito A., Maglio O., Nastri F., Lombardi A. (2024). Peptides and metal ions: A successful marriage for developing artificial metalloproteins. J. Pept. Sci..

[37] Kang X., Wang L., Liu B., Zhou S., Li Y., Yang S.-L., Yao R., Qiao L., Wang X., Gong W. (2024). Mechanically rigid metallopeptide nanostructures achieved by highly efficient folding. Nat. Synth..

[38] Bonczidai-Kelemen D., Tóth K., Fábián I., Lihi N. (2024). The role of the terminal cysteine moiety in a metallopeptide mimicking the active site of the NiSOD enzyme. Dalton Trans..

[39] Han J., Yan X., Yoon J. (2024). Self-assembled Peptide-based Biocatalyst. Peptide Self-Assembly and Engineering.

[40] Yao M., Dong S., Xu X. (2024). Asymmetric Carbene Transformations for the Construction of All-Carbon Quaternary Centers. Chem.—A Eur. J..

[41] Balhara R., Chatterjee R., Jindal G. (2024). Mechanism and stereoselectivity in metal and enzyme catalyzed carbene insertion into X–H and C (sp 2)–H bonds. Chem. Soc. Rev..

[42] Pan Y., Wang Y., Karmakar S., Sivaguru P., Liu Z. (2024). Catalytic alkane C–H functionalization by carbene insertion of unactivated C (sp3)–H bonds. Org. Chem. Front..

[43] Díaz-Requejo M.M., Pérez P.J. (2020). The TpxM Core in Csp3–H Bond Functionalization Reactions: Comparing Carbene, Nitrene, and Oxo Insertion Processes (Tpx = Scorpionate Ligand; M = Cu, Ag). Eur. J. Inorg. Chem..

[44] Rumo C., Stein A., Klehr J., Tachibana R., Prescimone A., Häussinger D., Ward T.R. (2022). An Artificial Metalloenzyme Based on a Copper Heteroscorpionate Enables sp3 C–H Functionalization via Intramolecular Carbene Insertion. J. Am. Chem. Soc..

[45] Jacobs L.M.C., Consol P., Chen Y. (2024). Drug Discovery in the Field of β-Lactams: An Academic Perspective. Antibiotics.

[46] Muñoz-Molina J.M., Belderrain T.R., Pérez P.J. (2019). Trispyrazolylborate coinage metals complexes: Structural features and catalytic transformations. Coord. Chem. Rev..

[47] Facchetti G., Rimoldi I. (2018). 8-Amino-5,6,7,8-tetrahydroquinoline in iridium(iii) biotinylated Cp* complex as artificial imine reductase. New J. Chem..

[48] Pellizzoni M., Facchetti G., Gandolfi R., Fusè M., Contini A., Rimoldi I. (2016). Evaluation of Chemical Diversity of Biotinylated Chiral 1,3-Diamines as a Catalytic Moiety in Artificial Imine Reductase. ChemCatChem.

[49] Liang A.D., Serrano-Plana J., Peterson R.L., Ward T.R. (2019). Artificial Metalloenzymes Based on the Biotin-Streptavidin Technology: Enzymatic Cascades and Directed Evolution. Acc. Chem. Res..

[50] Mukherjee P., Sairaman A., Deka H.J., Jain S., Mishra S.K., Roy S., Bhaumik P., Maiti D. (2024). Enantiodivergent synthesis of isoindolones catalysed by a Rh(III)-based artificial metalloenzyme. Nat. Synth..

[51] Zhao J., Bachmann D.G., Lenz M., Gillingham D.G., Ward T.R. (2018). An artificial metalloenzyme for carbene transfer based on a biotinylated dirhodium anchored within streptavidin. Catal. Sci. Technol..

[52] Robles V.M., Dürrenberger M., Heinisch T., Lledós A., Schirmer T., Ward T.R., Maréchal J.-D. (2014). Structural, Kinetic, and Docking Studies of Artificial Imine Reductases Based on Biotin–Streptavidin Technology: An Induced Lock-and-Key Hypothesis. J. Am. Chem. Soc..

[53] Natoli S.N., Hartwig J.F. (2019). Noble−Metal Substitution in Hemoproteins: An Emerging Strategy for Abiological Catalysis. Acc. Chem. Res..

[54] Key H.M., Dydio P., Clark D.S., Hartwig J.F. (2016). Abiological catalysis by artificial haem proteins containing noble metals in place of iron. Nature.

[55] Dydio P., Key H.M., Nazarenko A., Rha J.Y.-E., Seyedkazemi V., Clark D.S., Hartwig J.F. (2016). An artificial metalloenzyme with the kinetics of native enzymes. Science.

[56] Lemon C.M. (2023). Diversifying the functions of heme proteins with non-porphyrin cofactors. J. Inorg. Biochem..

[57] Pott M., Tinzl M., Hayashi T., Ota Y., Dunkelmann D., Mittl P.R.E., Hilvert D. (2021). Noncanonical Heme Ligands Steer Carbene Transfer Reactivity in an Artificial Metalloenzyme. Angew. Chem. Int. Ed..

[58] Gu Y., Natoli S.N., Liu Z., Clark D.S., Hartwig J.F. (2019). Site-Selective Functionalization of (sp3)C−H Bonds Catalyzed by Artificial Metalloenzymes Containing an Iridium-Porphyrin Cofactor. Angew. Chem. Int. Ed..

[59] Bloomer B.J., Natoli S.N., Garcia-Borràs M., Pereira J.H., Hu D.B., Adams P.D., Houk K.N., Clark D.S., Hartwig J.F. (2023). Mechanistic and structural characterization of an iridium-containing cytochrome reveals kinetically relevant cofactor dynamics. Nat. Catal..

[60] Mukherjee A., Roy S. (2024). Non-Electrostatic Basis for an Artificial Metalloenzyme Catalysis. bioRxiv.

[61] Huang X., Besset T., Jubault P., Couve-Bonnaire S. (2023). Fluorinated Substrates as Michael Acceptors Towards Fine Chemicals. Adv. Synth. Catal..

[62] Deehan T., Hellier P., Ladommatos N. (2024). The influence of Michael acceptors on the structural reactivity of renewable fuels. Sustain. Energy Fuels.

[63] Samanta B., Shankar Panda B., Mohapatra S., Nayak S. (2024). Current Developments in Michael Addition Reaction using Heterocycles as Convenient Michael Donors. Asian J. Org. Chem..

[64] Morita Y., Kubo H., Matsumoto R., Fujieda N. (2024). A thiopyridine-bound mirror-image copper center in an artificial non-heme metalloenzyme. J. Inorg. Biochem..

[65] Fujieda N., Ichihashi H., Yuasa M., Nishikawa Y., Kurisu G., Itoh S. (2020). Cupin Variants as a Macromolecular Ligand Library for Stereoselective Michael Addition of Nitroalkanes. Angew. Chem..

[66] Fujieda N., Nakano T., Taniguchi Y., Ichihashi H., Sugimoto H., Morimoto Y., Nishikawa Y., Kurisu G., Itoh S. (2017). A Well-Defined Osmium–Cupin Complex: Hyperstable Artificial Osmium Peroxygenase. J. Am. Chem. Soc..

[67] Tabares L.C., Daniel D.T., Vázquez-Ibar J.L., Kouklovsky C., Alezra V., Un S. (2023). Using the Noncanonical Metallo-Amino Acid [Cu(II)(2,2′-Bipyridin-5-yl)]-alanine to Study the Structures of Proteins. J. Phys. Chem. Lett..

[68] Lamartina C.W., Chartier C.A., Hirano J.M., Shah N.H., Rovis T. (2024). Crafting Unnatural Peptide Macrocycles via Rh(III)-Catalyzed Carboamidation. J. Am. Chem. Soc..

[69] Learte-Aymamí S., Martínez-Castro L., González-González C., Condeminas M., Martin-Malpartida P., Tomás-Gamasa M., Baúlde S., Couceiro J.R., Maréchal J.-D., Macias M.J. (2024). De Novo Engineering of Pd-Metalloproteins and Their Use as Intracellular Catalysts. JACS Au.

[70] Singh K., Kaur A., Goyal B., Goyal D. (2024). Harnessing the Therapeutic Potential of Peptides for Synergistic Treatment of Alzheimer’s Disease by Targeting Aβ Aggregation, Metal-Mediated Aβ Aggregation, Cholinesterase, Tau Degradation, and Oxidative Stress. ACS Chem. Neurosci..

[71] Wang C., Shao S., Li N., Zhang Z., Zhang H., Liu B. (2023). Advances in Alzheimer’s Disease-Associated Aβ Therapy Based on Peptide. Int. J. Mol. Sci..

[72] Fujieda N., Tonomura A., Mochizuki T., Itoh S. (2024). Asymmetric Michael addition catalysed by copper–amyloid complexes. RSC Adv..

[73] Kjaergaard C.H., Qayyum M.F., Wong S.D., Xu F., Hemsworth G.R., Walton D.J., Young N.A., Davies G.J., Walton P.H., Johansen K.S. (2014). Spectroscopic and computational insight into the activation of O_2_ by the mononuclear Cu center in polysaccharide monooxygenases. Proc. Natl. Acad. Sci. USA.

[74] Himes R.A., Barnese K., Karlin K.D. (2010). One is Lonely and Three is a Crowd: Two Coppers Are for Methane Oxidation. Angew. Chem. Int. Ed..

[75] Iacovino L.G., Pinzi L., Facchetti G., Bortolini B., Christodoulou M.S., Binda C., Rastelli G., Rimoldi I., Passarella D., Di Paolo M.L. (2021). Promising Non-cytotoxic Monosubstituted Chalcones to Target Monoamine Oxidase-B. ACS Med. Chem. Lett..

[76] Pellegrino S., Facchetti G., Contini A., Gelmi M.L., Erba E., Gandolfi R., Rimoldi I. (2016). Ctr-1 Mets7 motif inspiring new peptide ligands for Cu(i)-catalyzed asymmetric Henry reactions under green conditions. RSC Adv..

[77] Coffetti G., Moraschi M., Facchetti G., Rimoldi I. (2023). The Challenging Treatment of Cisplatin-Resistant Tumors: State of the Art and Future Perspectives. Molecules.

[78] Arnesano F., Natile G. (2021). Interference between copper transport systems and platinum drugs. Semin. Cancer Biol..

[79] Pellegrino S., Contini A., Gelmi M.L., Lo Presti L., Soave R., Erba E. (2014). Asymmetric Modular Synthesis of a Semirigid Dipeptide Mimetic by Cascade Cycloaddition/Ring Rearrangement and Borohydride Reduction. J. Org. Chem..

[80] Rimoldi I., Bucci R., Feni L., Santagostini L., Facchetti G., Pellegrino S. (2021). Exploring the copper binding ability of Mets7 hCtr-1 protein domain and His7 derivative: An insight in Michael addition catalysis. J. Pept. Sci..

[81] Mavlankar N.A., Maulik A., Pal A. (2024). Metal co-factors to enhance catalytic activity of short prion-derived peptide sequences. Pept. Catal. Incl. Catal. Amyloids.

[82] Tao K., Wu H., Adler-Abramovich L., Zhang J., Fan X., Wang Y., Zhang Y., Tofail S.A.M., Mei D., Li J. (2024). Aromatic short peptide architectonics: Assembly and engineering. Prog. Mater. Sci..

[83] Facchetti G., Vitoria J.G., Moraschi M., Bucci R., Abel A.C., Pieraccini S., Pellegrino S., Rimoldi I. (2023). Exploitation of Dimeric Cyclic Cysteine as Helix Inducer in Ultra-Short Peptides for Cu(II)-Catalyzed Asymmetric Michael Addition on Chalcones. Eur. J. Org. Chem..

[84] Oliva F., Bucci R., Tamborini L., Pieraccini S., Pinto A., Pellegrino S. (2019). Bicyclic pyrrolidine-isoxazoline γ amino acid: A constrained scaffold for stabilizing α-turn conformation in isolated peptides. Front. Chem..

[85] Heravi M.M., Zadsirjan V., Saedi P., Momeni T. (2018). Applications of Friedel–Crafts reactions in total synthesis of natural products. RSC Adv..

[86] Ohata J. (2024). Friedel–Crafts reactions for biomolecular chemistry. Org. Biomol. Chem..

[87] Haapalainen A.M., van Aalten D.M., Meriläinen G., Jalonen J.E., Pirilä P., Wierenga R.K., Hiltunen J.K., Glumoff T. (2001). Crystal structure of the liganded SCP-2-like domain of human peroxisomal multifunctional enzyme type 2 at 1.75 Å resolution. J. Mol. Biol..

[88] Van Stappen C., Deng Y., Liu Y., Heidari H., Wang J.-X., Zhou Y., Ledray A.P., Lu Y. (2022). Designing Artificial Metalloenzymes by Tuning of the Environment beyond the Primary Coordination Sphere. Chem. Rev..

[89] Jarvis A.G., Obrecht L., Deuss P.J., Laan W., Gibson E.K., Wells P.P., Kamer P.C.J. (2017). Enzyme Activity by Design: An Artificial Rhodium Hydroformylase for Linear Aldehydes. Angew. Chem. Int. Ed..

[90] Doble M.V., Jarvis A.G., Ward A.C.C., Colburn J.D., Götze J.P., Bühl M., Kamer P.C.J. (2018). Artificial Metalloenzymes as Catalysts for Oxidative Lignin Degradation. ACS Sustain. Chem. Eng..

[91] Kuckhoff T., Brewster R.C., Ferguson C.T.J., Jarvis A.G. (2023). Reactivity Tuning of Metal-Free Artificial Photoenzymes through Binding Site Specific Bioconjugation. Eur. J. Org. Chem..

[92] Pandya C., Sivaramakrishna A. (2024). Organoruthenium-bipyridyl complexes—A platform for diverse chemistry and applications. Coord. Chem. Rev..

[93] Hu Z., Liang J., Su T., Zhang D., Li H., Gao X., Yao W., Song X. (2023). Minimizing the Anticodon-Recognized Loop of Methanococcus jannaschii Tyrosyl-tRNA Synthetase to Improve the Efficiency of Incorporating Noncanonical Amino Acids. Biomolecules.

[94] Klemencic E., Brewster R.C., Ali H.S., Richardson J.M., Jarvis A.G. (2024). Using BpyAla to generate copper artificial metalloenzymes: A catalytic and structural study. Catal. Sci. Technol..

[95] Gutiérrez de Souza C., Bersellini M., Roelfes G. (2020). Artificial Metalloenzymes based on TetR Proteins and Cu(II) for Enantioselective Friedel-Crafts Alkylation Reactions. ChemCatChem.

[96] Drienovská I., Scheele R.A., Gutiérrez de Souza C., Roelfes G. (2020). A Hydroxyquinoline-Based Unnatural Amino Acid for the Design of Novel Artificial Metalloenzymes. ChemBioChem.

[97] Roelfes G. (2022). Supramolecular Assembly of DNA—And Protein-Based Artificial Metalloenzymes. Supramolecular Catalysis.

[98] Bos J., Browne W.R., Driessen A.J.M., Roelfes G. (2015). Supramolecular Assembly of Artificial Metalloenzymes Based on the Dimeric Protein LmrR as Promiscuous Scaffold. J. Am. Chem. Soc..

[99] Chordia S., Narasimhan S., Lucini Paioni A., Baldus M., Roelfes G. (2021). In Vivo Assembly of Artificial Metalloenzymes and Application in Whole-Cell Biocatalysis. Angew. Chem. Int. Ed..

[100] Saptal V.B., Ruta V., Bajada M.A., Vilé G. (2023). Single-Atom Catalysis in Organic Synthesis. Angew. Chem. Int. Ed..

[101] Kment Š., Bakandritsos A., Tantis I., Kmentová H., Zuo Y., Henrotte O., Naldoni A., Otyepka M., Varma R.S., Zbořil R. (2024). Single Atom Catalysts Based on Earth-Abundant Metals for Energy-Related Applications. Chem. Rev..

[102] Fang J., Chen Q., Li Z., Mao J., Li Y. (2023). The synthesis of single-atom catalysts for heterogeneous catalysis. Chem. Commun..

[103] Guo Z., Hong J., Song N., Liang M. (2024). Single-Atom Nanozymes: From Precisely Engineering to Extensive Applications. Acc. Mater. Res..

[104] Zhang L., Ren Y., Liu W., Wang A., Zhang T. (2018). Single-atom catalyst: A rising star for green synthesis of fine chemicals. Natl. Sci. Rev..

[105] Li X., Cao Y., Xiong J., Li J., Xiao H., Li X., Gou Q., Ge J. (2023). Enzyme-metal-single-atom hybrid catalysts for one-pot chemoenzymatic reactions. Chin. J. Catal..

[106] Zhu J., Zhang Y., Lu D., Zare R.N., Ge J., Liu Z. (2013). Temperature-responsive enzyme–polymer nanoconjugates with enhanced catalytic activities in organic media. Chem. Commun..

[107] Cao S., Xu P., Ma Y., Yao X., Yao Y., Zong M., Li X., Lou W. (2016). Recent advances in immobilized enzymes on nanocarriers. Chin. J. Catal..

[108] Nikoshvili L.Z., Matveeva V.G. (2023). Recent Progress in Pd-Catalyzed Tandem Processes. Catalysts.

[109] Reissig H.-U. (2024). Introduction to the Chemistry of Donor–Acceptor Cyclopropanes: A Historical and Personal Perspective. Donor Acceptor Cyclopropanes in Organic Synthesis.

[110] Talele T.T. (2016). The “Cyclopropyl Fragment” is a Versatile Player that Frequently Appears in Preclinical/Clinical Drug Molecules. J. Med. Chem..

[111] Sun M.-R., Li H.-L., Ba M.-Y., Cheng W., Zhu H.-L., Duan Y.-T. (2021). Cyclopropyl Scaffold: A Generalist for Marketed Drugs. Mini Rev. Med. Chem..

[112] Hoffman G.R., Olson M.G., Schoffstall A.M., Estévez R.F., Van den Eynde V., Gillman P.K., Stabio M.E. (2023). Classics in Chemical Neuroscience: Selegiline, Isocarboxazid, Phenelzine, and Tranylcypromine. ACS Chem. Neurosci..

[113] Li H., Cheng J. (2024). 2-Phenylcyclopropylmethylamine (PCPMA) as a privileged scaffold for central nervous system drug design. Bioorganic Med. Chem. Lett..

[114] Tinoco A., Wei Y., Bacik J.-P., Carminati D.M., Moore E.J., Ando N., Zhang Y., Fasan R. (2019). Origin of High Stereocontrol in Olefin Cyclopropanation Catalyzed by an Engineered Carbene Transferase. ACS Catal..

[115] Chandgude A.L., Fasan R. (2018). Highly Diastereo- and Enantioselective Synthesis of Nitrile-Substituted Cyclopropanes by Myoglobin-Mediated Carbene Transfer Catalysis. Angew. Chem. Int. Ed..

[116] Bajaj P., Sreenilayam G., Tyagi V., Fasan R. (2016). Gram-Scale Synthesis of Chiral Cyclopropane-Containing Drugs and Drug Precursors with Engineered Myoglobin Catalysts Featuring Complementary Stereoselectivity. Angew. Chem. Int. Ed..

[117] Carminati D.M., Fasan R. (2019). Stereoselective Cyclopropanation of Electron-Deficient Olefins with a Cofactor Redesigned Carbene Transferase Featuring Radical Reactivity. ACS Catal..

[118] Melngaile R., Videja M., Kuka J., Kinens A., Zacs D., Veliks J. (2023). Synthetic Access to Fluorocyclopropylidenes. Org. Lett..

[119] Zhang G., McCorvy J.D., Shen S., Cheng J., Roth B.L., Kozikowski A.P. (2019). Design of fluorinated cyclopropane derivatives of 2-phenylcyclopropylmethylamine leading to identification of a selective serotonin 2C (5-HT2C) receptor agonist without 5-HT2B agonism. Eur. J. Med. Chem..

[120] Pons A., Delion L., Poisson T., Charette A.B., Jubault P. (2021). Asymmetric Synthesis of Fluoro, Fluoromethyl, Difluoromethyl, and Trifluoromethylcyclopropanes. Acc. Chem. Res..

[121] Yamani K., Pierre H., Archambeau A., Meyer C., Cossy J. (2020). Asymmetric Transfer Hydrogenation of gem-Difluorocyclopropenyl Esters: Access to Enantioenriched gem-Difluorocyclopropanes. Angew. Chem. Int. Ed..

[122] Pellissier H. (2021). Recent developments in enantioselective zinc-catalyzed transformations. Coord. Chem. Rev..

[123] Schaus L., Das A., Knight A.M., Jimenez-Osés G., Houk K.N., Garcia-Borràs M., Arnold F.H., Huang X. (2023). Protoglobin-Catalyzed Formation of cis-Trifluoromethyl-Substituted Cyclopropanes by Carbene Transfer. Angew. Chem. Int. Ed..

[124] Ren X., Chandgude A.L., Carminati D.M., Shen Z., Khare S.D., Fasan R. (2022). Highly stereoselective and enantiodivergent synthesis of cyclopropylphosphonates with engineered carbene transferases. Chem. Sci..

[125] Villada J.D., Majhi J., Lehuédé V., Hendricks M.E., Neufeld K., Tona V., Fasan R. (2024). Biocatalytic Strategy for the Highly Stereoselective Synthesis of Fluorinated Cyclopropanes. Angew. Chem. Int. Ed..

[126] Sikandar S., Zahoor A.F., Ghaffar A., Anjum M.N., Noreen R., Irfan A., Munir B., Kotwica-Mojzych K., Mojzych M. (2023). Unveiling the Chemistry and Synthetic Potential of Catalytic Cycloaddition Reaction of Allenes: A Review. Molecules.

[127] Gregg T.M., Farrugia M.K., Frost J.R. (2009). Rhodium-mediated enantioselective cyclopropanation of allenes. Org. Lett..

[128] Hasegawa Y., Cantin T., Decaens J., Couve-Bonnaire S., Charette A.B., Poisson T., Jubault P. (2022). Catalytic Asymmetric Syntheses of Alkylidenecyclopropanes from Allenoates with Donor-Acceptor and Diacceptor Diazo Reagents. Chem.—A Eur. J..

[129] Lindsay V.N.G., Fiset D., Gritsch P.J., Azzi S., Charette A.B. (2013). Stereoselective Rh2(S-IBAZ)4-Catalyzed Cyclopropanation of Alkenes, Alkynes, and Allenes: Asymmetric Synthesis of Diacceptor Cyclopropylphosphonates and Alkylidenecyclopropanes. J. Am. Chem. Soc..

[130] Bloomer B.J., Joyner I.A., Garcia-Borràs M., Hu D.B., Garçon M., Quest A., Ugarte Montero C., Yu I.F., Clark D.S., Hartwig J.F. (2024). Enantio- and Diastereodivergent Cyclopropanation of Allenes by Directed Evolution of an Iridium-Containing Cytochrome. J. Am. Chem. Soc..

[131] Maglio O., Chino M., D’Alonzo D., Leone L., Nastri F., Lombardi A. (2023). Peptide-based Artificial Metalloenzymes by Design. Peptide and Protein Engineering for Biotechnological and Therapeutic Applications.

[132] Zozulia O., Korendovych I.V. (2020). Semi-Rationally Designed Short Peptides Self-Assemble and Bind Hemin to Promote Cyclopropanation. Angew. Chem. Int. Ed..

[133] Villarino L., Splan K.E., Reddem E., Alonso-Cotchico L., Gutiérrez de Souza C., Lledós A., Maréchal J.-D., Thunnissen A.-M.W.H., Roelfes G. (2018). An Artificial Heme Enzyme for Cyclopropanation Reactions. Angew. Chem. Int. Ed..

